# Prognostic Significance of Molecular Analysis of Peritoneal Fluid for Patients with Gastric Cancer: A Meta-Analysis

**DOI:** 10.1371/journal.pone.0151608

**Published:** 2016-03-17

**Authors:** Kai Deng, Hong Zhu, Mo Chen, Junchao Wu, Renwei Hu, Chengwei Tang

**Affiliations:** 1 Department of Gastroenterology, West China Hospital, Sichuan University, Chengdu, 610041, China; 2 Department of Abdominal Cancer, West China Hospital, Sichuan University, Chengdu, 610041, China; 3 Department of Critical Care Medicine, Chengdu Second People’s Hospital, Chengdu, 610041, China; National Cancer Center, JAPAN

## Abstract

**Background:**

Accurately distinguishing serosal invasion in patients with gastric cancer (GC) prior to surgery can be difficult. Molecular analysis of peritoneal fluid (MAPF) for free cancer cells with higher sensitivity than other methods; however, its prognostic value for GC remains controversial, precluding its application in clinical practice.

**Methods:**

PubMed, EMBASE and other databases were systematically searched. Thirty-one studies were eligible for the meta-analysis. Hazard ratios (HRs) and 95% confidence intervals (CIs) were pooled for overall survival (OS), disease-free survival (DFS) and peritoneal recurrence-free survival (PRF).

**Results:**

The current meta-analysis focused on patients with GC and negative cytological diagnoses. The results showed that positive MAPF status (MAPF^+^) led to poorer prognoses for OS (HR 2.59, 95% CI 1.99–3.37), DFS (HR 4.92, 95% CI 3.28–7.37) and PRF (HR 2.81, 95% CI 2.12–3.72) compared with negative MAPF status (MAPF^-^). Moreover, among the patients with GC who received curative treatment, the MAPF^+^ patients had poorer prognoses for OS (HR 3.27, 95% CI 2.49–4.29), DFS (HR 3.90, 95% CI 2.74–5.57) and PRF (HR 5.45, 95% CI 3.70–8.03). A meta-analysis of multivariate-adjusted HRs demonstrated that MAPF^+^ status was an independent prognostic factor for patients with GC who underwent curative treatment (OS: HR 2.19, 95% CI 1.47–3.28; PRF: HR 3.44, 95% CI 2.01–5.87). Using the identical target genes (CEA, CEA/CK20) as molecular markers, the patients with GC who were MAPF^+^ had significantly worse prognoses for OS (CEA: HR 3.03, 95% CI 2.29–4.01; CEA/CK20: HR 4.24, 95% CI 2.42–7.40), DFS (CEA: HR 3.99, 95% CI 2.24–7.12; CEA/CK20: HR 4.31, 95% CI 1.49–2.48) and PRF (CEA: HR 4.45, 95% CI 2.72–7.31; CEA/CK20: HR 6.46, 95% CI 3.62–11.55) than the patients who were MAPF^-^.

**Conclusion/Significance:**

The above results demonstrate that MAPF could be a prognostic indicator for patients with GC who have a negative cytological diagnosis and/or are receiving curative treatment. MAPF could provide clinicians with additional prognostic information that could aid in developing individualized treatment plans prior to surgery. The widely used target genes CEA, CEA/CK20 were confirmed to be valuable MAPF markers for predicting the prognosis of GC.

## Introduction

Gastric cancer (GC) remains one of the most common causes of cancer-related mortality worldwide. Approximately one million patients are diagnosed with GC annually; however, the available treatments are unsatisfactory[1]. To date, surgical resection has been the preferred method for curative treatment of GC. As technology has rapidly advanced, minimally invasive procedures have been introduced for GC. The small incisions associated with these procedures leave less scar tissue and help reduce postoperative pain. Additionally, patients who undergo minimally invasive procedures recover more quickly than patients who undergo conventional surgery or extended resection. Accuracy in detecting small tumors that have invaded the abdominal cavity and in predicting the prognosis prior to surgery is difficult to attain, especially during the early and middle stages of GC. Minimal amounts of residual cancer could result in tumor recurrence and poor prognosis, which may elevate the risk of recurrence and result in a need to undergo addittional operations [2,3,4,5]; as such, patients may be warry in opting for minimally invasive surgery. Therefore, the ability to pre- and postoperatively predict occult micrometastasis would be extremely valuable for developing individualized treatment plans and in choosing an adjuvant chemotherapy (AC), which may be of additional benefit to patients with GC. Once the high-risks factors for peritoneal micrometastasis are identified, intraperitoneal chemotherapy (IPC) can be performed to minimize recurrence after surgery [6].

The peritoneal fluid (PF) surrounding the outer wall containing the gastrointestinal organs may contain trace amounts of tumor cells. Peritoneal cytology was developed to identify GC patients with poor prognoses[7,8,9]; however, when the proportion of exfoliative tumor cells is too low to be diagnosed by a pathologist, the positive rate of peritoneal cytology is limited. Polymerase chain reaction (PCR) offers far greater sensitivity than exfoliative cytology and has therefore been increasingly utilized to detect trace amounts of tumor cells. Over the past decade, many studies have shown that the detection of tumor mRNAs (e.g., CEA, CK20, CK19 and MMP-7) in the PF using PCR is associated with adverse outcomes in patients with GC[10,11,12,13]. A systematic review recently confirmed the diagnostic value of CEA mRNA in predicting peritoneal recurrence of GC[14]. Accurately identifying the risk of incurring a poor prognosis (e.g., recurrence and mortality) is valuable for clinicians who must balance the benefits and losses of administering AC to patients with GC. The prognostic value of PF analysis in patients with GC has varied between studies. Pecqueux M, et al. recently published a review focused on the identification of free intraperitoneal tumor cells and indicated the prognostic value of this approach for patients with GC[15]. However, no detailed analysis of the prognostic value of molecular analysis of PF (MAPF) has been performed. Utilizing PCR to detect free cancer cells has the advantage of high sensitivity, especially in cases with negative cytological diagnoses based on assessments of PF[13]. The risks of a poor prognosis for patients found to possess free tumor cells based on molecular analysis of PF relative to those not found to possess free tumor cells vary greatly among studies. Therefore, it is necessary to conduct a comprehensive study to precisely estimate the prognostic value of utilizing MAPF to evaluate patients with GC to accelerate the clinical application of this method. In the current study, we performed a meta-analysis of published studies to obtain a detailed estimation of the prognostic value of MAPF.

## Methods

### Search Strategy

A systematic search was performed to identify all relevant literature. The PubMed and EMBASE databases were searched with the following index formulas (explained in detail in S2 Table): ((((("Polymerase Chain Reaction"[All field]) OR ("Reverse Transcriptase Polymerase Chain Reaction"[All field])) AND ((minim* resid*) OR ("Flow Cytometry"[Mesh]) OR ("cytology" [Subheading]) OR ("DNA"[Mesh]) OR ("RNA"[Mesh]) OR (shedd* cell*) OR (tumo* cell*) OR (cancer* cell*) OR (neoplas* cell*))) AND ("Stomach Neoplasms"[Mesh] AND English[lang] AND "humans"[Mesh] NOT Case Reports[ptyp] NOT Letter[ptyp] NOT Review[ptyp] NOT Comment[ptyp])) AND (("Ascitic Fluid"[Mesh]) OR (peritone* wash*) OR (peritone* cavi* water) OR (peritoneal* lavage*) OR (efflus*))) AND ((prognos*) OR (risk*) OR (survival*) OR (recurren*) OR (factor*) OR (marker*) OR (biomarker*) OR (relevan*) OR (role*)); (prognos* OR risk* OR survival* OR recurren* OR factor* OR marker* OR biomarker* OR relevan* OR role*) AND (ascitic* AND fluid* OR (peritone* AND wash*) OR (peritone* AND cavi* AND water) OR (peritoneal* AND lavage*) OR efflus*) AND ((gastr* OR stomac*) AND (cancer* OR carcinom* OR neoplas* OR tumo*)) AND ((polymerase AND chain AND reaction) OR (reverse AND transcriptase AND polymerase AND chain AND reaction) AND ((minim* AND resid*) OR (flow AND cytometry) OR cytology OR dna OR rna OR (shedd* AND cell*) OR (tumo* AND cell*) OR (cancer* AND cell*) OR (neoplas* AND cell*))) AND (([article]/lim OR [article in press]/lim OR [conference abstract]/lim OR [conference paper]/lim) AND [english]/lim AND [humans]/lim). We also manually searched the Journal of Clinical Oncology (JCO), the American Society of Clinical Oncology (ASCO) annual meeting abstracts and the Cochrane Library. Cited references from selected studies were also scanned to identify additional relevant studies. All potentially relevant papers were obtained and evaluated in detail. The searches for published articles were augmented with results from unpublished reports. The search was updated on August 10, 2015.

### Study Selection

**Fig 1:**
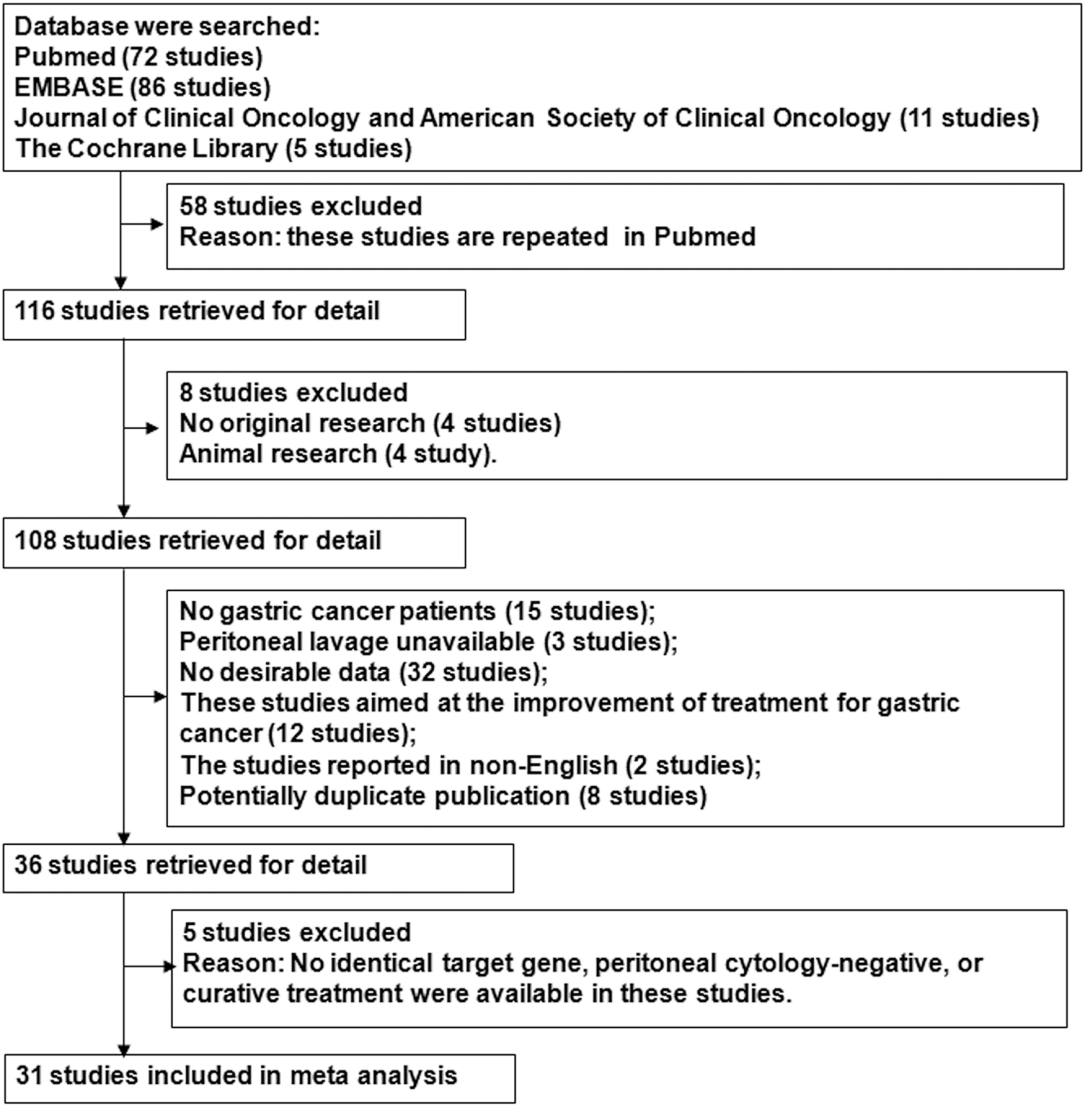
Selection of included studies.

All articles identified in the literature search were subsequently screened for eligibility using the following inclusion criteria: (1) all patients were histologically diagnosed with GC; (2) analysis of PF (peritoneal washing, peritoneal lavage, peritoneal effusion or ascitic fluid) was performed using PCR; (3) prognostic analysis of MAPF status [(presence of free tumor cells (MAPF^+^) vs. absence of free tumor cells (MAPF^-^)] was performed with hazard ratios (HRs), Kaplan-Meier survival curves or log-rank tests in accordance with the MAPF status required for each article; and (4) only studies (should be not less than three) assessing identical target genes, patients with negative peritoneal cytology, or curative treatment were included to control between-studies variability. The exclusion criteria were as follows: (1) animal research; (2) non-original research (e.g., reviews, comments, letters, and case reports); (3) insufficient data to estimate HRs for mortality, recurrence or peritoneal recurrence; (4) trials aimed at improving the treatment of GC; and (5) studies not reported in English. A flow chart representing the study selection process is listed in Fig 1. If two data sets overlapped or were duplicated, the article with more information was retained. Twelve of the articles that were identified were reported by the same research team. Because the reported results might have been obtained from the same series of patients, eight studies were excluded for potentially overlapping datas[16,17,18,19,20,21,22,23], and four studies with more patients or more information from the same research teams were retained in the final analysis[24,25,26,13]. Five studies[27,28,24,29,30] without identical target genes, negative peritoneal cytology, or the use of curative treatments were eliminated to control between-studies variability.

### Data Abstraction

The studies selected in the initial search were independently assessed by two researchers (Kai Deng and Hong Zhu) for their adherence to the inclusion and exclusion criteria. Standardized methods were applied to each of the included studies, from which the following information was extracted: first author, publication year, country, study period, target genes, definition of MAPF status, eligible cases per group, age, tumor stage, follow-up period, peritoneal cytology, surgical treatment, HR and corresponding 95% confidence interval (CI), and covariates adjusted by multivariate Cox regression analysis. The HRs and /or 95% CIs were retrieved either directly or indirectly. If a HR and its 95% CI were not presented directly, they were estimated from the data provided in the articles by using previously reported statistical methods (explained in S1 File)[31]. The 9-star Newcastle-Ottawa Scale (NOS) was used to assess the quality of the included studies (non-randomized studies)[32].

### Statistical Analyses

In the meta-analysis, overall survival (OS), disease-free survival (DFS) and peritoneal recurrence-free survival (PRF) were used as outcome endpoints for patients with GC, and HRs and 95% CIs were pooled. OS, DFS and PRF were calculated starting at diagnosis until total death, recurrence, peritoneal recurrence or last follow-up visit. In the meta-analysis, the HR represented the risk of adverse outcomes for MAPF^+^ patients compared with MAPF^-^ patients. As a mixture of log-rank tests and multivariate Cox regression analyses were used in the included studies, their HRs were combined. The statistical heterogeneity among the included studies was assessed by using the *I*^2^ statistic (significance at a 10% level)[33]. If heterogeneity was found, the following approach was used to understand its origin: meta-regression was applied to explore the potential sources of heterogeneity, and subgroup analyses were performed to reduce the heterogeneity; if these methods failed, a random-effects model was used. If the heterogeneity was insignificant, a fixed effects model was used with the inverse variance method [34]. When distinct heterogeneity existed, a random-effects model was used with the DerSimonian-Laird method [35]. The meta-regression and subgroup analyses were used to identify potential sources of heterogeneity within the characteristics of the studies. In the meta-regression, these characteristics (e.g., curative treatment, cytology status, area, positive rate of eligible cases, adjuvant chemotherapy, publication year, NOS score, multivariate vs. univariate analysis, eligible cases) were used as covariates. Moreover, in each meta-analysis, potential publication bias was graphically assessed using Begg’s funnel plot and statistically estimated using Egger’s test (significance at 5% level)[36]. If publication bias was found, the “trim and fill” method was used to assess the stability of the estimated effects. Statistical analysis was conducted using Stata 12.0 (StataCorp LP, College Station, TX). All *P* values were two-sided, and significance levels were set at 0.05 (except for the *I*^2^ statistic). The results of each study are shown by a point estimate of the HR (the size of the square is proportional to the weight) and the corresponding 95% CI.

## Results

### Baseline Characteristics of the Included Studies

The systematic literature search yielded 31 studies, including 3224 patients with GC for inclusion in the final analysis (Fig 1 and Table 1). Based on survival analyses, all of the patients in 15 of the included studies showed negative conventional pathological cytology of the PF (cytology^-^)[37,38,39,40,26,41,42,10,43,13,44,45,46,47,48]. In 14 of the studies, curative treatments were applied to all patients who were included in the survival analyses [37,42,49,10,11,50,51,52,26,44,43,45,41,53]. The identical target genes (CEA[37,42,49,11,25,51,40,54,55,53], CEA/CK20[56,46,10,50,57,44,43,58]) were chosen as molecular biomarkers of free cancer cells in 25 of the studies. The baseline characteristics of the included studies are summarized in Table 1. After assessing the risk of bias for the individual studies, 13 studies had a high risk of bias[56,42,46,50,59,44,60,61,48,45,58,53,47] and 18 studies had a low risk of bias[37,38,51,54,39,40,52,12,49,26,41,25,11,55,10,43,13,57] (S3 Table online). Thirteen of the included studies confirmed that the MAPF status was confirmed as an independent prognostic factor in multivariate Cox regression analysis. HRs for OS, DFS and PRF could be extracted from 20, 8 and 18 studies, respectively (Table 1). In accordance with the NOS scores, the quality scores of the eligible studies ranged from 6 to 9 (mean, 7.7; S4 Table online).

**Table 1:** Baseline characteristics of selected studies.

Author, publication year	Target genes	Eligible sample* (MAPF^+^/MAPF^-^)	Follow-up period (months)	Adjuvant treatment	HRs (95% CIs) in terms of OS, DFS and/or PRF; adjusted variables
Fujii S, 2002[56]	CEA	27/22	16–60	NA	PRF^b^ 25.46 (1.61–402.98);
Fujiwara Y, 2014[37]	CEA	DFS and PRF: 55/49^d^; OS: 59/51^e^	< 50	NA	DFS^a^ 2.30 (1.25–4.22); PRF^a^ 6.85 (2.48–18.88); OS^a^ 2.38 (1.26–4.50).
Fukumoto Y, 2006[42]	CEA	4/16^d^^,^ ^e^	< 40	NA	DFS^a^ 3.20 (1.55–6.62);
Hara M, 2007[49]	CEA	19/107^d^	22.4 (4.0–38.2)	NA	OS^c^ 2.81 (1.03–7.63), adjusted for histology, depth of tumor invasion, lymph node metastasis.
Ishii T, 2004[10]	CEA	OS and PRF: 5/46^d^^,^ ^e^	< 60	NA	OS^a^ 3.57 (1.42–8.98); PRF^a^ 5.10 (1.55–16.74).
Ito S, 2005[11]	CEA	Retrospective Study OS, PRF: 55/142^d^; Prospective study OS and PRF: 20/66^d^	Retrospective study: 38; Prospective study: 30 (21–50).	Adjuvant Chemotherapy (AC)	Retrospective study: OS^a^ 7.94 (3.50–18.01), PRF ^a^ 22.34 (5.08–98.19); Prospective study: OS^c^ 1.79 (1.13–2.85), PRF ^c^ 3.99 (1.80–8.84). Both were adjusted for tumor size, histological type, serosal invasion, and lymph node metastasis.
Katsuragi K, 2007[12]	CEA or CK20	15/65^d^	32	NA	OS^c^ 9.9 (2.3–43.0), adjusted for tumor depth, stage, lymph node metastasis, lymphatic invasion, venous invasion, histological type.
Kodera Y, 2006[25]	CEA	OS: 98/176; PRF:70/172^f^	83.2 (61–143.5)	NA	OS^c^ 1.89 (1.17–3.07), adjusted for nodal status, serosal invasion, cytology, hepatic metastasis, surgical resection, and peritoneal deposits; PRF^c^ 1.57 (1.07–2.29), adjusted for nodal status, serosal invasion, and cytology.
Li Z, 2014[51]	CEA	40/76 ^d^	36	AC	OS^a^ 3.85 (2.11–7.01); PRF^c^ 3.04 (1.47–6.26), adjusted for TGF-beta1, CA125, MMP-7, CK20, and PLC.
Masahiro Horikawa, 2011[46]	CEA	OS and PRF: 41/106^e^	37 (7–68)	NA	OS^c^ 1.92 (1.16–3.13), PRF^c^ 1.75 (1.09–2.86). Both were adjusted for CD44 mRNA, tumor size, lymph node metastasis, and TNM stage.
Miyagawa K, 2008[52]	RegIV	30/47^d^	> 24	NA	OS^a^ 2.92 (0.67–12.77);
Mori K, 2007[26]	Two or more of CK20, FABP1, MUC2, TFF1, TFF2, MASPIN, GW112, PRSS4, TAC-STD1 or CEA	6/50^d^^,^ ^e^	> 23.3	NA	DFS^a^ 6.50 (2.05–20.61);
Nakanishi H, 1999[44]	CEA	29/53^d^^,^ ^e^	< 37	NA	OS^a^ 4.99 (1.79–13.91);
Okada K, 2012[40]	CEA	10/26 ^e^	< 100	NA	OS^a^ 2.10 (1.16–3.80);
Oyama K, 2004[43]	CEA	OS, DFS and PRF: 30/133 ^d^^,^ ^e^	27.1 (1.4–51.6)	AC, intraperitoneal Chemotherapy (IPC)	DFS^a^ 8.50 (3.75–19.25); OS^c^ 6.10 (1.15–32.46), PRF^c^ 14.49 (1.35–155.20). Both were adjusted for sex, depth of tumor invasion, lymph node metastasis, histological type, and tumor size.
Rossi Del Monte S, 2012[60]	CEA or CK20	OS and DFS: 21/6	17 (1–27)	NA	OS^c^ 39.60 (1.01–1551.60), DFS^c^ 29.10 (1.01–837.60). Both were adjusted for histology, depth of invasion, nodal status, stage at primary diagnosis, and IF evaluation.
Satoh Y, 2012[39]	CK20, FABP1 or MUC2	12/40 ^e^	20.9 (0.9–33.6)	NA	PRF^a^ 9.09 (2.16–38.31);
Sugita Y, 2003[61]	CEA or CK20	59/70	NA	NA	PRF^b^ 23.73 (3.28–171.56);
Takebayashi K, 2014[45]	CEA or CK20	80/22^d^^,^ ^e^	50	NA	PRF^a^ 11.26 (1.355–93.58);
Tamura N, 2007[41]	CEA or CK20	OS: 28/112^e^; PRF: 19/93^d^	26 (18–65)	NA	OS^a^ 3.43 (1.86–6.33); PRF^a^ 11.79 (3.89–35.75);
Tamura S, 2014[54]	CEA	OS: 51/89; PRF: 50/74^f^	< 50	AC	OS^c^ 1.80 (0.90–3.70), PRF^c^ 3.40 (1.40–9.50). Both were adjusted for Age, gender, T stage, pN, histopathology, and CK20
Tokuda K, 2003[58]	CEA	30/106	NA	NA	OS^b^ 10.60 (4.19–26.80); PRF^b^ 53.00 (7.29–385.11).
Wang JY, 2005[55]	CEA	11/29	25 (18–32)	NA	PRF^c^ 4.08 (1.75–9.53), adjusted for cytology and pCEA levels.
Wong J, 2012[53]	CEA	13/72^d^	35	AC	OS^a^ 4.80 (1.28–18.01).
Yoneda A, 2014[38]	CK19	OS, DFS and PRF: 12/31^e^	39 (6–51)	NA	OS^a^ 16.31 (1.13–234.53), DFS^a^ 18.85 (2.23–159.56), PRF ^a^ 8.57 (1.47–49.89).
Jeon CH, 2014[50]	CEA	38/79^d^	36	NA	DFS ^b^ 4.99 (1.89–13.15)
Yonemura Y, 2001[13]	MMP-7	OS and PRF: 17/108^e^	28.8 (8–47)	NA	OS^c^ 2.46 (1.07–5.66), adjusted for lymph node metastasis, serosal invasion, lymphatic invasion, vessel invasion, histological type, and cytology. PRF ^b^ 2.36 (1.62–3.45)
Lee SR, 2013[59]	CEA or CK20	28/86	26 (17–35)	NA	PRF^c^ 3.67 (1.69–7.96), adjusted for tumor location, tumor size, T stage, N stage and perineural invasion.
Yabusaki N 2015[47]	pZEB1	18/54^e^	41.9 (1–106)	NA	DFS^a^^,^^e^ 3.84 (1.38–10.64);
Takata A 2014[48]	CEA or CK20	16/88^e^	18.2	AC	DFS^c^^,^^e^ 3.49 (1.14–10.70), adjusted for age, sex, histology, neoadjuvant chemotherapy, pT stage, and pN stage.
Nakabayashi K 2015[57]	CEA	36/92	40	NA	OS^a^ 4.56(1.63–12.74).

*, Ineligible cases reported in the original articles were excluded;

^a^, HR and 95% CI values were extracted from survival curves;

^b^, HR and 95% CI values were estimated from variance and the *P*-values;

^c^, HR and 95% CI values were estimated from a multivariate Cox proportional hazards regression analysis;

^d^: Curative surgery was performed in all patients;

^e^: All cases were negative by cytology;

^f^: The patients with peritoneal metastasis were excluded from the survival analysis; Abbreviations: MAPF: molecular analysis of peritoneal fluid; OS, overall survival; DFS, disease-free survival; PRF, peritoneal recurrence-free survival; NA, not available; HR, hazard ratio; CI, confidence interval.

### Negative Cytological Status

**Fig 2:**
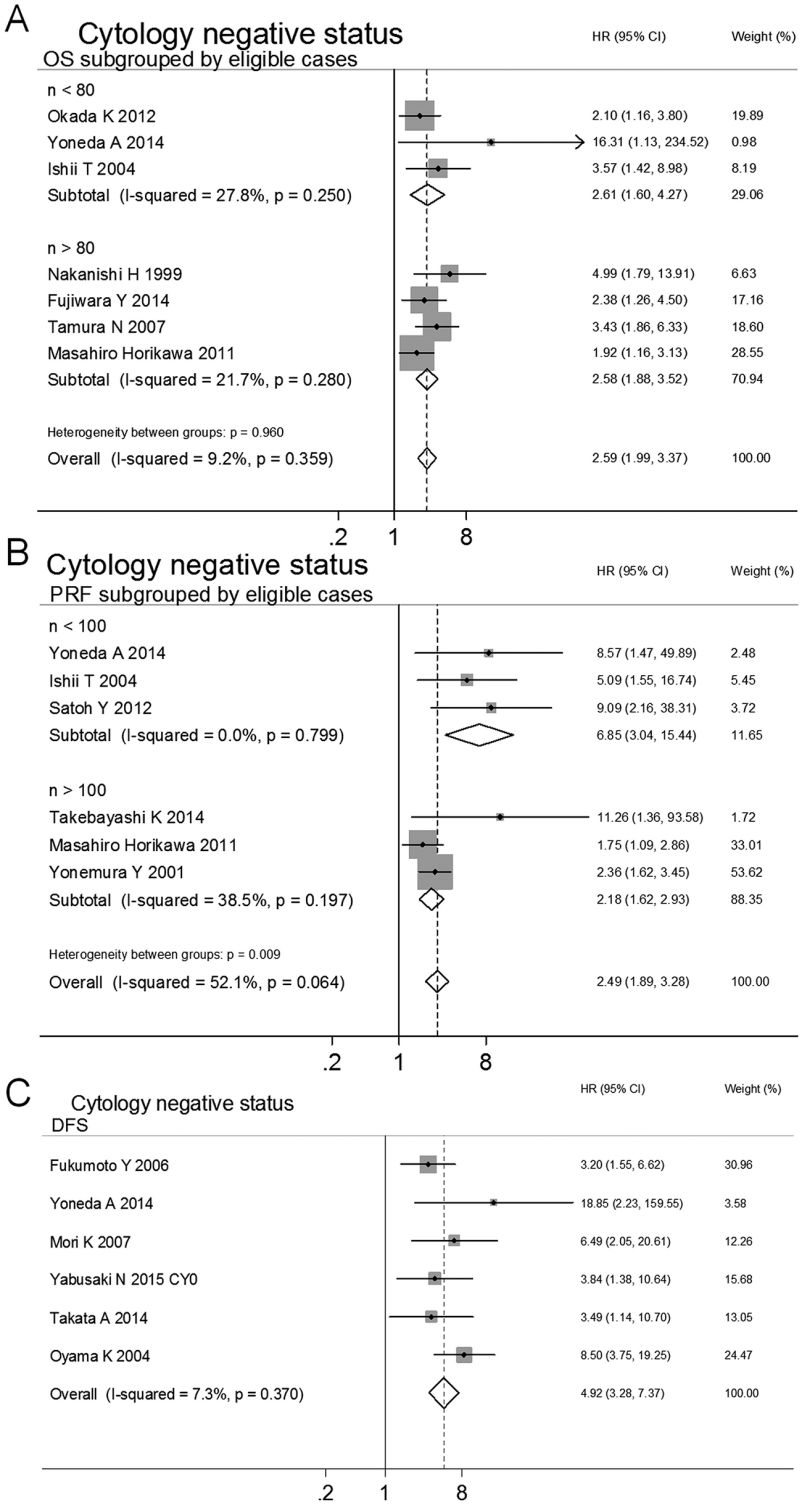
Subgroup analyses and meta-analyses show that the MAPF has prognostic value for OS (A), DFS (B) and PRF (C) in patients with GC with negative cytology.

**Fig 3:**
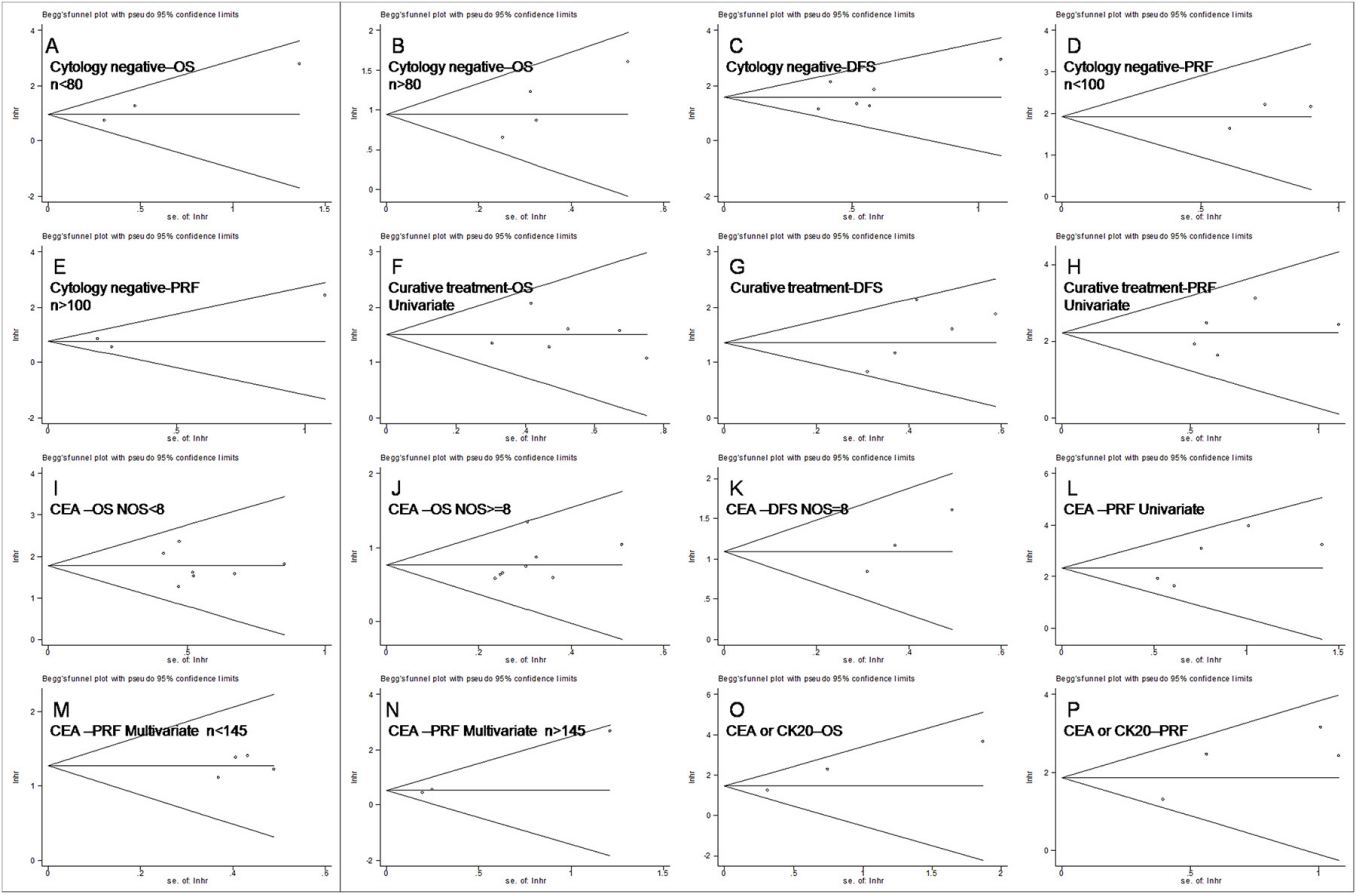
Funnel plots to evaluate the publication biases of OS (A, B, F, I, J and O), DFS (C, G, and K) and PRF (D, E, H, L, M, N and P) in subgroup analyses.

To control for variability generated from PF cytology status, studies evaluating cytology^-^ patients were pooled. Fifteen studies were selected to evaluate the prognostic effects of various endpoints (OS[37,46,10,44,40,41,38], DFS[42,26,43,48,47,38] and PRF[46,10,39,45,38,13]) for GC. MAPF^+^ patients had poor prognoses for OS (HR 2.59, 95%CI 1.99–3.37, n = 7, *I*^2^ = 9.2%, *P*_Q_ = 0.359), DFS (HR 4.92, 95%CI 3.28–7.37, n = 6, *I*^2^ = 7.3%, *P*_Q_ = 0.370) and PRF (HR 2.81, 95%CI 2.12–3.72, n = 6, *I*^2^ = 35.3%, *P*_Q_ = 0.172), as shown in Table 2. Although no significant between-studies heterogeneity was observed in the above meta-analyses, publication bias was found in Begg’s funnel plot analysis and Egger’s tests of OS and PRF (Table 2). In the meta-regression analyses, no association was found between estimated effects and study characteristics (e.g., curative treatment, cytological status, positive eligible case rate, area, year published, NOS score, uni- vs. multivariate analyses, and eligible cases), as shown in Table 2. In the subgroup analyses of eligible cases, the publication bias disappeared, and the prognostic value of MAPF for GC was again identified for OS (eligible cases < 80, HR 2.61, 95% CI 1.60–4.27, n = 3, *I*^2^ = 27.8%, *P*_Q_ = 0.250; eligible cases > 80, HR 2.58, 95% CI 1.88–3.52, n = 4, *I*^2^ = 21.7%, *P*_Q_ = 0.280) and PRF (eligible cases < 100, HR 6.85, 95% CI 3.04–15.44, n = 3, *I*^2^ = 0.0%, *P*_Q_ = 0.799; eligible cases > 100, HR 2.18, 95% CI 1.62–2.93, n = 3, *I*^2^ = 38.5%, *P*_Q_ = 0.197) (Table 2, Figs 2 and 3A–3E). Inter-suggroup heterogeneity was analyzed in the subgroup analyses (OS: eligible cases < 80 vs. eligible cases > 80, *P*_between-groups_ = 0.960; PRF: eligible cases < 100 vs. eligible cases > 100, *P*_between-groups_ = 0.009), as shown in Table 2. The stability of estimated effects on OS, DFS and PRF was validated in the "trim and fill" analyses (Table 2).

**Table 2:** Subgroup analysis of the associations between the MAPF status and adverse outcomes (OS, DFS and PRF) in patients with GC.

	HR (95% CI), *I*^2^(n), *p*_Q_	Publication bias^a^	Stability of estimated effect^b^	Heterogeneity between subgroups (*p*_between-groups_)	Meta-regression analysis^e^ (*p*_regression_)
**CEA**					
**OS**	3.03 (2.29–4.01), 52.9%(15), 0.008	0.033, 0.007	stable	-	Multivariate/Univariate (0.009), NOS (0.003), extraction (0.043)
* * ***AC vs*. *no-AC***				0.638	
AC	3.33 (1.97–5.71), 63.9%(6), 0.016	0.348, 0.209	stable		
no-AC	2.89 (2.05–4.06), 48.9%(9), 0.047	0.007, 0.007	stable		
* * ***NOS<8 vs*. *NOS> = 8***				< 0.001	
* * NOS<8	5.92(4.02–8.73), 0.0% (7), 0.728	0.881, 0.602	stable		-
* * NOS> = 8	2.14 (1.74–2.62), 0.0%(8), 0.622	0.048, 0.277	stable		-
**DFS**	3.99 (2.24–7.12), 56.2%(4), 0.077	0.174, 0.233	stable	-	NOS (0.154), AC (0.154)
* * ***AC vs*. *no-AC***				0.025	
* * AC	8.50 (3.75–19.25), -(1), -	-	-		-
* * no-AC	2.97 (1.95–4.52), 0.0%(3), 0.403	0.117, 0.077	stable		-
* * ***NOS = 7 vs*. *NOS = 8***				0.025	
* * NOS = 7	8.50 (3.75–19.25), -(1), -	-	-		-
* * NOS = 8	2.97 (1.95–4.52), 0.0%(3), 0.403	0.117, 0.077	stable		-
* ***PRF**	4.45 (2.72–7.31), 71.3%(12), <0.001	0.004, <0.001	stable	-	Multivariate/Univariate (0.008), NOS (0.048), extraction (0.035)
* * ***AC vs*. *no-AC***				0.015	
* * AC	4.24 (2.74–6.57), 42.2%(5), 0.140	0.142, 0.085	stable		
* * no-AC	4.13 (2.11–8.11), 76.5%(7), <0.001	0.051, 0.001	stable		
* * ***Univariate vs*. *Multivariate***				< 0.001	
* * Univariate	10.28 (5.47–19.29), 33.9%(5), 0.195	0.327, 0.120	stable		-
* * Multivariate	2.19 (1.72–2.79), 50.1%(7), 0.061	0.051, 0.002	stable		n (0.043), NOS (0.072)
***n<145 vs*. *n>145***				0.004	
n < 145	3.57 (2.37–5.38), 0.0%(4), 0.946	0.497, 0.628	stable		-
n > 145	1.70 (1.26–2.28), 39.7%(3), 0.190	0.117, 0.004	stable		-
**CEA or CK20**					
**OS**	4.24 (2.42–7.40), 37.0%(3), 0.205	0.602, 0.145	stable	-	-
**DFS**	4.31 (1.49–12.48), 27.4%(2), 0.241	-, -	stable	-	-
***AC vs*. *no-AC***				0.241	
AC	3.49 (1.14–10.69), -(1), -	-	-		
no-AC	29.10 (1.01–837.60), -(1), -	-	-		
**PRF**	6.46 (3.62–11.55), 41.3%(4), 0.164	0.497, 0.184	stable	-	-
**Cytology negative status**^c^					
**OS**	2.59 (1.99–3.37), 9.2%(7), 0.359	0.011, 0.016	stable	-	None
***n<80 vs*. *n>80***				0.960	
n < 80	2.61 (1.60–4.27), 27.8%(3), 0.250	0.117, 0.135	stable		-
n > 80	2.58 (1.88–3.52), 21.7%(4), 0.280	0.174, 0.157	stable		-
**DFS**	4.92 (3.28–7.37), 7.3%(6), 0.370	0.348, 0.329	stable	-	None
***AC vs*. *no-AC***				0.371	
AC	6.24 (3.22–12.07), 36.9%(2), 0.208	0.317, -	stable		
no-AC	4.26 (2.55–7.11), 0.3%(4), 0.390	0.042, 0.035	stable		
**PRF**	2.81 (2.12–3.72), 35.3%(6), 0.172	0.039, <0.001	stable	-	n (0.042)
***n<100 vs*. *n>100***				0.009	
n < 100	6.85 (3.04–15.44), 0.0%(3), 0.799	0.602, 0.398	stable		-
n >100	2.18 (1.62–2.93), 38.5%(3), 0.197	0.602, 0.485	stable		-
**Curative treatment**^d^					
**OS**	3.27 (2.49–4.29), 45.3%(9), 0.067	0.677, 0.074	stable	-	Multivariate/Univariate (0.075)
***AC vs*. *no-AC***				0.067	
AC	3.74 (1.85–7.54), 73.6%(4), 0.010	0.497, 0.275	stable		
no-AC	4.00 (2.43–6.57), 0.0%(5), 0.669	0.624, 0.466	stable		-
***Univariate vs*. *Multivariate***				0.009	
Univariate	4.54 (3.15–6.56), 0.0%(6), 0.742	0.851, 0.933	stable		-
Multivariate	2.19 (1.47–3.28), 60.4%(3), 0.080	0.117, 0.812	stable		-
**DFS**	3.90 (2.74–5.57), 47.8%(5), 0.105	0.142, 0.152	stable	-	None
**PRF**	5.45 (3.70–8.03), 34.1%(7), 0.168	0.072, 0.035	stable	-	Multivariate/Univariate (0.058)
***Univariate vs*. *Multivariate***				0.014	
Univariate	9.05 (5.16–15.86), 0.0%(5), 0.578	0.624, 0.457	stable		-
Multivariate	3.44 (2.01–5.87), 0.0%(2), 0.618	-	stable		-

^a^: Potential publication bias was assessed using Begg’s funnel plot and Egger’s test in each meta-analysis;

^b^: If publication bias was observed, the trim and filled method was used to access the stability of the estimated effect;

^c^: All cases were negative by peritoneal fluid cytology;

^d^: Curative treatment was performed in all patients.

^e^: In the meta-regression, select characteristics (e.g., curative treatment, cytology status, area, positive rate of eligible cases, adjuvant chemotherapy, publication year, NOS, multivariate vs. univariated analysis, eligible cases) were used as covariates. NOS, Newcastle Ottawa Scale; AC: gastric cancer patients who underwent adjuvant chemotherapy were included; no-AC: gastric cancer patients who underwent adjuvant chemotherapy were not included. *p*_Q_: Q statistic *p*-value; *p*_between-groups_: p-value for heterogeneity between subgroups; *p*_regression_: the *p*-value for meta-regression; None, no characteristics (curative treatment, cytology status, positive rate of eligibale cases, adjuvant chemotherapy, area, publication year, NOS score, multivariate vs. univariated analysis, eligible cases) were found in the meta-regression analysis.

To explore the potential variability associated with the use of adjuvant chemotherapy (AC), the following subgroup analyses were performed to combine HRs from studied evaluating patients with GC with or without AC (AC vs. no-AC). In a pooled analysis of the AC and no-AC groups, the predicted reccurence in cytology^-^ GC patients according to MAPF was validated in terms of DFS (all HRs and 95% CIs > 1, in Table 2). Moreover, no association was found between pooled HRs and AC in the meta-regression analysis, indicating that the poor prognoses observed in the MAPF^+^ patients with GC was independent from the use of chemotherapy (all *P* > 0.05), as shown in Table 2.

Collectively, the above results demonstrated that the MAPF had prognostic value in terms of OS, DFS and PRF for cytology^-^ patients with GC. For this subset of patients, a MAPF^+^ status represented a greater risk for peritoneal recurrence and mortality.

### Curative Treatment

**Fig 4:**
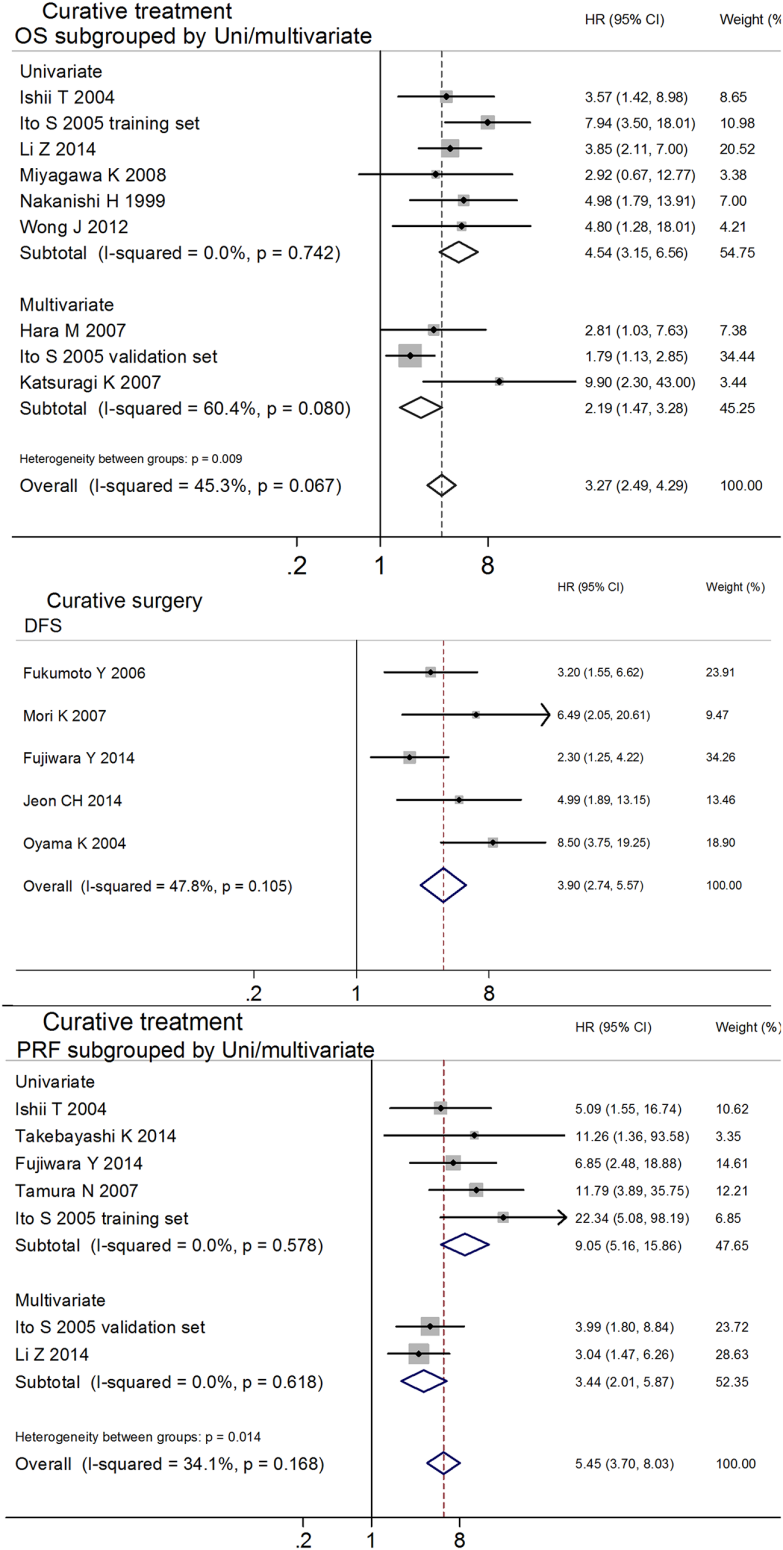
Subgroup analyses and meta-analyses show that the MAPF status has prognostic effects on OS (A), DFS (B) and PRF (C) in patients with GC who received curative treatment.

To control treatment variability, studies concerning patients who received curative treatments were pooled. Fourteen studies were selected to evaluate the prognostic effects of various endpoints (OS[49,10,11,51,52,44,53], DFS[37,42,50,26,43] and PRF[37]) for GC. Two independent clinical trials were reported in one study[11], and the HRs and 95% CIs were calculated separately. The analysis indicated that MAPF^+^ patients showed poor prognosis for OS (HR 3.27, 95%CI 2.49–4.29, n = 9, *I*^2^ = 45.3%, *P*_Q_ = 0.067), DFS (HR 3.90, 95%CI 2.74–5.57, n = 5, *I*^2^ = 47.8%, *P*_Q_ = 0.105) and PRF (HR 5.45, 95%CI 3.70–8.03, n = 7, *I*^2^ = 34.1%, *P*_Q_ = 0.168), as shown in Table 2. Significant between-studies heterogeneity was observed in the combined analyses of OS, and publication bias was detected in the meta-analysis of PRF (Table 2). In the meta-regression analyses of OS and PRF, the potential associations identified between the estimated effects and uni-/multivariate analyses attracted our attention (OS: uni-/multivariate *P*_regression_ = 0.075; PRF: uni-/multivariate *P*_regression_ = 0.058), as shown in Table 2. In the uni- and multivariate subgroup analyses, the significant heterogeneity and publication bias respectively disappeared, and the prognostic value of MAPF for GC was confirmed for OS (univariate group: HR 4.54, 95% CI 3.15–6.56, n = 6, *I*^2^ = 0.0%, *P*_Q_ = 0.742; multivariate group: HR 2.19, 95% CI 1.47–3.28, n = 3; univariate group vs. multivariate group, *P*_between-groups_ = 0.009) and PRF (univariate group: HR 9.05, 95% CI 5.16–15.86, n = 5, *I*^2^ = 0.0%, *P*_Q_ = 0.578; multivariate group: HR 3.44, 95% CI 2.01–5.87, n = 2, *I*^2^ = 0.0%, *P*_Q_ = 0.618; univariate group vs. multivariate group, *P*_between-groups_ = 0.014; Table 2, Figs 3F–3H and 4). The stability of the estimated effects on OS, DFS and PRF was validated in the "trim and fill" analyses (Table 2).

To minimize the potential impact from AC, the following subgroup analyses were divided into AC and no-AC groups to assess the pooled HRs, In the pooled analysis of AC and no-AC groups, the poor survival of MAPF^+^ patients who underwent curative treatment was confirmed in terms of OS (all HRs and 95% CIs > 1), as shown in Table 2. Moreover, no association was found between pooled HRs and AC in our meta-regression analysis, indicating that the poor prognoses in the MAPF^+^ patients with GC are independent from chemotherapy (all *P* > 0.05), as shown in Table 2.

The above results demonstrated that MAPF has prognostic value in terms of OS, DFS and PRF for patients with GC who receive curative treatment. MAPF can provide additional prognostic information for patients with GC prior to surgery and help clinicians develop individualized treatment plans (e.g., minimally invasive therapy, extended resection, or adjuvant therapy).

### CEA, CEA/CK20

**Fig 5:**
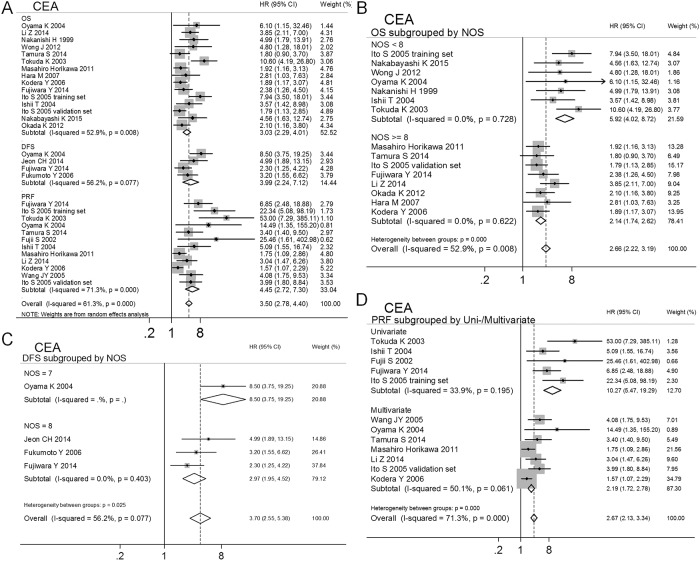
Subgroup analyses using CEA as a target gene for MAPF to predict GC prognosis. A) Subgroup analysis by various endpoints (OS, DFS and PRF). Significant heterogeneity disappeared in the subgroup analysis of NOS score with respect to OS (B) and DFS (C). D) The univariate and multivariate analyses used in the studies were a major source of heterogeneity in terms of PRF.

To control for the heterogeneity caused by the use of different target genes in the examined studies, the studies that assessed identical target genes were pooled. CEA was widely used as a target gene for MAPF of patients with GC, being examined in 18 studies. Meta-analyses of these studies were performed to calculate the prognostic effects of various endpoints (OS[37,49,10,11,25,51,57,44,40,43,54,58,53], DFS[37,42,50,43] and PRF[56,37,10,11,25,51,43,54,58,55]) for GC. Two independent clinical trials were reported in one study[11], and the HRs and 95% CIs were calculated separately. The results indicated poor prognoses in terms of OS (HR 3.03, 95%CI 2.29–4.01, n = 15, *I*^2^ = 52.9%, *P*_Q_ = 0.008), DFS (HR 3.99, 95%CI 2.24–7.12, n = 4, *I*^2^ = 56.2%, *P*_Q_ = 0.077) and PRF (HR 2.67, 95%CI 2.13–3.34, n = 12, *I*^2^ = 71.3%, *P*_Q_ < 0.001) for MAPF^+^ patients, as shown in Table 2 and Fig 2A. Meta-regression analyses were performed to identify the major sources of heterogeneity. The results showed that some of the characteristics of the included studies were associated with the estimated effects (OS: uni-/multivariate *P*_regression_ = 0.009, NOS *P*_regression_ = 0.003; PRF: uni-/multivariate *P*_regression_ = 0.008, NOS *P*_regression_ = 0.048; shown), as shown in Table 2. Significant heterogeneity between subgroups was observed in the following pooled analyses divided by study characteristics (OS: NOS < 8 vs. NOS ≥ 8, *P*_between-groups_ < 0.001; DFS: NOS = 7 vs. NOS = 8, *P*_between-groups_ = 0.025; PRF: univariate vs. multivariate *P*_between-groups_ < 0.001; multivariate group for PRF: eligible cases < 145 vs. eligible cases > 145 *P*_between-groups_ = 0.004), as shown in Fig 5 and Table 2. In the subgroup analyses based on NOS score, uni-/multivariate analyses, or eligible cases, significant heterogeneity and publication bias disappeared, and the prognostic value of MAPF for GC was confirmed for OS (NOS < 8 group: HR 5.92, 95% CI 4.02–8.72, n = 7, *I*^2^ = 0.0%, *P*_Q_ = 0.728; NOS ≥ 8 group: HR 2.14, 95% CI 1.74–2.62, n = 8, *I*^2^ = 0.0%, *P*_Q_ = 0.531), DFS (NOS = 7 group: HR 8.50, 95% CI 3.75–19.25, n = 1; NOS = 8 group: HR 2.97, 95% CI 1.95–4.52, n = 3, *I*^2^ = 0.0%, *P*_Q_ = 0.403) and PRF (univariate group: HR 10.28, 95% CI 5.47–19.29, n = 5, *I*^2^ = 33.9%, *P*_Q_ = 0.195; multivariate group: eligible cases < 145 subgroup, HR 3.57, 95% CI 2.37–5.38, n = 4, *I*^2^ = 0.0%, *P*_Q_ = 0.946 vs. eligible cases > 145 subgroup, HR 1.70, 95% CI 1.26–2.28, n = 3, *I*^2^ = 39.7%, *P*_Q_ = 0.190) (Table 2, Figs 3T–3M and 5). The predictive effects of MAPF were tested in "trim and fill" analyses (Table 2). Although the pooled HRs for OS, DFS and PRF were influenced by various study characteristics, including NOS score, use of uni- vs. multivariate analysis, and eligible cases), all pooled HRs were greater than 1. These results indicated that MAPF using CEA as a target gene had prognostic value for patients with GC, regardless of NOS score, use of uni- vs. multivariate analysis, and eligible cases.

**Fig 6:**
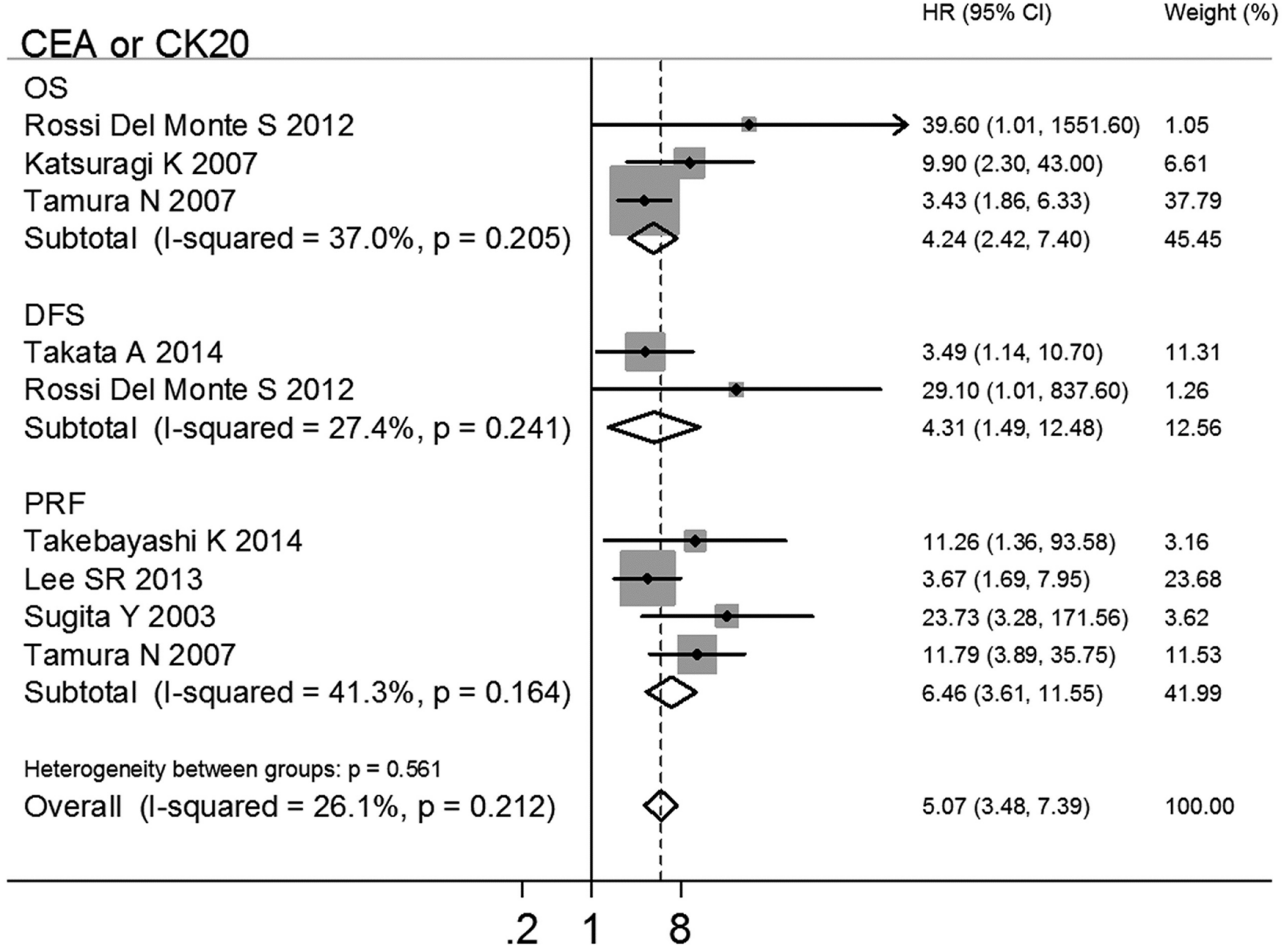
Subgroup analysis (by various endpoints) of the studies using CEA/CK20 as the target genes for MAPF to predict GC prognosis.

Seven studies used CEA/CK20 as target genes for MAPF. These studies were analyzed to assess the prognostic effects of various endpoints (OS[12,60,41], DFS[60,48] and PRF[59,61,45,41]) for GC. The results showed that MAPF^+^ patients had poor prognoses in terms of OS (HR 4.24, 95%CI 2.42–7.40, n = 3, *I*^2^ = 37.0%, *P*_Q_ = 0.205), DFS (HR 4.31, 95%CI 1.49–12.48, n = 2, *I*^2^ = 27.4%, *P*_Q_ = 0.241) and PRF (HR 6.46, 95%CI 3.62–11.55, n = 4, *I*^2^ = 41.3%, *P*_Q_ = 0.164), as shown in Table 2 and Fig 6. No heterogeneity or publication bias was observed in the pooled analysis (Table 2 and Fig 3P and 3Q). The stability of the estimated effects was validated in the "trim and fill" analyses (Table 2).

These results indicated that MAPF using CEA/CK20 as target genes had prognostic value for OS, DFS and PRF for patients with GC. When identical target genes (CEA, CEA/CK20) were detected, MAPF^+^ patients with GC had a higher risk of peritoneal recurrence and mortality.

To minimize the potential impact of AC, the following pooled analyses using CEA or CEA/CK20 as target genes were divided into AC and no-AC groups for the purpose of combining HRs. In these pooled analyses, higher recurrence and poorer survival were observed in MAPF^+^ patients with respect to OS, DFS and PRF (all HRs and 95% CIs > 1), as shown in Table 2. Moreover, no association was found between the pooled HRs and AC in meta-regression analysis, indicating that the poor prognosis of MAPF^+^ patients with GC is independent from the use of chemotherapy (all *P* > 0.05), an shown in Table 2. Currently, IPC is recommended during surgery to improve the poor prognosis of patients with GC[6]. However, one study showed that IPC did not increase the survival of GC patients with macroscopic peritoneal metastasis (9 cases with IPC vs. 8 cases without IPC, *P* = 0.701)[43].

## Discussion

Currently, the accurate prediction of small cancerous invasions in patients with GC prior to treatment is difficult. Conventional PF cytology is primarily used to detect free tumor cells and to predict serosal invasion and/or peritoneal dissemination. It has been demonstrated that the presence of free tumor cells in PF is associated with poor prognosis[15]. Therefore, positive PF cytology results indicate adverse outcomes for patients with GC. However, there are several disadvantages associated with this approach, limiting its application for prognostic prediction in patients with of GC. First, very few exfoliative tumor cells are present in PF in patients with GC until an end-stage is reached. This limited number of cells hinders the early detection of serosal invasion and/or peritoneal dissemination and decreases the clinical value of exfoliative cytology for prognostic prediction. Second, exfoliative cytology diagnoses are made by a pathologist who relies on cell morphology alone. Thus, a pathological diagnosis may be missed or inaccurate without the inclusion of histological analysis. In addition, experienced pathologists are needed for cytopathology to be accurate in detecting free tumor cells in the PF.

MAPF, which has the advantage of high sensitivity for detecting trace quantities of cancer cells, was developed to address the above-listed problems. A previous meta-analysis indicated that detection of CEA mRNA is a more sensitive method for detecting peritoneal recurrence than peritoneal lavage cytology[14]. In recent decades, many articles have been published validating the prognostic value of MAPF for patients with GC[12,26,13]. However, various target genes (e.g., CEA, CK20 and CK19) and endpoints have been reported in different studies, decreasing the ease of translating this method into the clinic. Therefore, we performed the current meta-analysis to confirm the HR for poor prognosis in GC patients that is associated with MAPF^+^ status.

MAPF offers complementary value to clinicians for predicting the prognosis of cytology^-^ patients with GC prior to surgery due to the following advantages. First, MAPF using select molecular markers may be more sensitive than peritoneal cytology in detecting free cancer cells. Even in cytology^-^ patients with GC, a MAPF^+^ status increases the risk of a poor prognosis by over twofold (Table 2 and Fig 2)[37,38,39,40,26,41,42,10,43,13,44,45,46,47,48]. For patients who receive curative treatment, the MAPF is useful for predicating peritoneal recurrence and mortality (Table 2 and Fig 4). This suggests that the MAPF^+^ patients who undergo curative treatment may benefit more from AC, particularly IPC, during surgery. It is widely accepted that IPC has positive effects in preventing peritoneal recurrence and improving survival[62], although at least one study has reported that IPC was of no benefit with respect to survival in GC patients with macroscopic peritoneal metastasis (9 cases with IPC vs. 8 cases without IPC, *P* = 0.701)[43]. This phenomenon might be explained by the small sample size and advanced pathological stage examined in the referenced study. Second, MAPF can be easily standardized and repeated in clinical laboratories. Additionally, MAPF can be conducted using conventional PCR techniques that are economical, technically easily and rapid. These advantages illustrate that the MAPF test has potential clinical value in making prognostic predictions for patients with GC prior to surgery.

Despite the substantial diversity in sample sizes, endpoints, target genes, treatment modalities and cytological findings among the included studies, the current meta-analysis increased the number of patients recruited on the basis of similar endpoints and overcame certain difficulties that are encountered in single studies. The large sample size used in this meta-analysis strengthens its statistical power and narrows its 95% CIs. To control the heterogeneity caused by variations in cytology status, treatment modalities and target genes, only studies examining cytology^-^ patients, curative treatments, or identical target genes were included in the current meta-analysis. The subgroup analyses of different endpoints indicated that the MAPF^+^ patients with GC had increased risks of mortality, recurrence and peritoneal recurrence compared to MAPF^-^ patients. In the meta-analysis that controlled for curative treatment, negative cytology status and identical target genes (CEA, CEA/CK20), the association between poor prognosis and the MAPF^+^ status persisted (Table 2). Increased risks of peritoneal recurrence and morality were observed for the MAPF^+^ patients who received curative treatment; these relationships were confirmed in the subsequent meta-analysis of multivariate-adjusted HRs (Fig 4A and 4C and Table 2). These results warn that certain types of surgery (e.g., endoscopic resection, function-preserving surgery, and others) should be performed with caution in MAPF^+^ patients with GC. AC may be required for MAPF^+^ patients undergoing curative treatment. The poor prognosis of MAPF^+^ patients was validated in all subgroup analyses performed in this work. Therefore, MAPF may be a promising approach to assess prognosis in the future.

Most of the pooled HRs from the AC group were higher than those from the no-AC group with respect to OS (CEA: 3.33 vs. 2.89; curative treatment: 3.74 vs. 4.00), DFS (CEA: 8.50 vs. 2.97; CEA/CK20: 3.49 vs. 29.10; cytology negative status: 6.24 vs. 4.26) and PRF (CEA: 4.24 vs. 4.13) (Table 2). In most of studies mentioned AC, it was recommed to GC patient undergo surgery depending on the clinical judgment. Tumor stage in the patients receiving AC was potentially poorer than that in patients not receiving AC. It can be reasonably inferred that a higher number of patients undergoging AC were MAPF^+^ vs. MAPF^-^, which may explain the enhancements observed in the pooled HRs from the AC group. After adjusting for the use of AC, the prognostic value of MAPF for DFS persisted in cytology^-^ patients (HR 3.49, 95% CI 1.14–10.70)[48]. The pooled HRs from the multivariate group were lower than those from the univariate group (CEA: PRF 2.19 vs.10.28; curative treatment: OS 2.19 vs. 4.54, PRF 3.44 vs. 9.05), as shown in Table 2. This can be easily explained by the adjustment for more variables reducing the confounding factors and thereby decreasing the estimated effect. In the pooled analysis of groups with lower NOS scores, the combined HRs were higher than those from groups with higher NOS scores (CEA: OS 5.92 vs. 2.14, DFS 8.50 vs. 2.97, in Table 2). This result can be explained by the better control of selection bias in the relatively more rigorous research. The pooled HRs from the studies with greater numbers of eligible cases were lower than those with lower numbers of eligible cases (CEA: PRF 3.57 vs. 1.70 in multivariate group; cytology negative status: OS 2.61 vs. 2.58, PRF 6.85 vs. 2.18), as shown in Table 2. The relatively low values of the pooled HRs may be the result of the large sample size enhancing the statistical power and minimizing the potential bias. Despite the fingding that the pooled HRs were slightly affected by AC, multivariate analysis, NOS score, and eligible case, the value of MAPF for prognostic prediction persisted throughout all of the pooled analyses and subgroup analyses.

Although the MAPF status can provide pretreatment prognostic information, PF cannot be collected as easily as peripheral blood. However, PF can be easily acquired by abdominal paracentesis or intraoperative peritoneal lavage, which is generally accepted by patients with GC, as it is minimally invasive. Therefore, the MAPF may be an acceptable examination method for clinicians trying to accurately assess prognosis and design an individualized treatment plans ot benefit patients with GC before initiating therapy.

Some deficiencies exist in this meta-analysis. First, the included studies covered a long time span (1998–2015), increasing the likelihood that differences in study characteristics existed from one institution to another. Despite the fact that different sample sizes, sample times, detection methods, target genes, treatments modalities, follow-up periods and ethnicities were used in the 31 studies, the interference factors may have become randomly balanced between the corresponding study arms. The meta-regression analysis found that some of the pooled HRs were influenced by study characteristics (uni- vs. multivariate analysis, NOS score and eligible cases), as shown in Table 2. Subgroup analyses based on these study characteristics were performed to eliminate the between-studies heterogeneity and to evaluate the HRs of various subgroups (all HRs >1, *P* < 0.05, Table 2). Second, the prognostic effects of the MAPF status were repeatedly confirmed through subgroup analyses and validated in “trim and fill” analyses, yet some potential confounders might have weakened the HR estimate. We cannot completely exclede the presence of prognosis-related confounding factors in the estimated HR. Although the subgroup analyses based on multivariate-adjusted HRs were performed to minimize potential confounders, the prognostic value of MAPF persisted with respect to OS (CEA: HR 1.95, 95% CI 1.53–2.50; CEA/CK20: 11.98, 95% CI 3.07–46.66; cytology negative status: HR 1.92, 95% CI 1.17–3.15; curative treatment: HR 2.94, 95% CI 1.27–6.85), DFS (CEA/CK20: HR 4.31, 95% CI 1.49–12.48; cytology negative status: HR 3.49, 95% CI 1.14–10.69;) and PRF (CEA: HR 2.60, 95% CI 1.77–3.83; CEA/CK20: HR 3.67, 95% 1.69–7.96; cytology negative status: HR 1.75, 95% CI 1.08–2.84; curative treatment: HR 3.44, 95% CI 2.01–5.87)(Table 2 and Figs 4 and 5).

## Conclusions

In summary, the current meta-analyses supported that MAPF status is a prognostic factor for patients with GC. The MAPF status can also be a prognostic predictor for patients with GC with negative cytology and can provide additional prognostic information to clinicians to aid in the development of individualized treatment plans prior to surgery. Although the molecular markers for MAPF were not unified, the widely used target genes (CEA, CEA/CK20) were confirmed to be valuable markers for predicting the prognosis of GC. Therefore, the MAPF aid in the accurate assessment of the prognosis for patients with GC, especially for easily underestimated cases.

## Supporting Information

S1 Fig PRISMA 2009 flow diagram of this meta-analysis. (DOC)

S1 File Detailed explanation for extraction of HR from survival analysis. (DOC)

S1 Table PRISMA 2009 checklist for this meta-analysis. (DOC)

S2 Table Search results from relevant articles. (DOC)

S3 Table Assessment of risk of bias. (DOC)

S4 Table Newcastle-Ottawa Scale (NOS) scores for assessing study quality. (DOC)

## References

[1] Jemal A, Bray F, Center MM, Ferlay J, Ward E, Forman D. Global cancer statistics. CA Cancer J Clin 2011;61: 69–90. 10.3322/caac.20107 21296855

[2] Abe S, Oda I, Nakajima T, Suzuki H, Nonaka S, Yoshinaga S, et al A case of local recurrence and distant metastasis following curative endoscopic submucosal dissection of early gastric cancer. Gastric Cancer 2015;18: 188–192. 10.1007/s10120-014-0341-7 24477418

[3] Fujii H, Ishii E, Tochitani S, Nakaji S, Hirata N, Kusanagi H, et al Lymph node metastasis after endoscopic submucosal dissection of a differentiated gastric cancer confined to the mucosa with an ulcer smaller than 30 mm. Dig Endosc 2015;27: 159–161. 10.1111/den.12261 24684669

[4] Song J, Lee HJ, Cho GS, Han SU, Kim MC, Ryu SW, et al Recurrence following laparoscopy-assisted gastrectomy for gastric cancer: a multicenter retrospective analysis of 1,417 patients. Ann Surg Oncol 2010;17: 1777–1786. 10.1245/s10434-010-0932-4 20151217

[5] Tong JH, Sun Z, Wang ZN, Zhao YH, Huang BJ, Li K, et al Early gastric cancer with signet-ring cell histologic type: risk factors of lymph node metastasis and indications of endoscopic surgery. Surgery 2011;149: 356–363. 10.1016/j.surg.2010.07.006 20727560

[6] Kuramoto M, Shimada S, Ikeshima S, Matsuo A, Yagi Y, Matsuda M, et al Extensive intraoperative peritoneal lavage as a standard prophylactic strategy for peritoneal recurrence in patients with gastric carcinoma. Ann Surg 2009;250: 242–246. 10.1097/SLA.0b013e3181b0c80e 19638909

[7] Hayes N, Wayman J, Wadehra V, Scott DJ, Raimes SA, Griffin SM. Peritoneal cytology in the surgical evaluation of gastric carcinoma. Br J Cancer 1999;79: 520–524. 10027323 10.1038/sj.bjc.6690081 PMC2362407

[8] Koga S, Kaibara N, Iitsuka Y, Kudo H, Kimura A, Hiraoka H. Prognostic significance of intraperitoneal free cancer cells in gastric cancer patients. J Cancer Res Clin Oncol 1984;108: 236–238. 6470030 10.1007/BF00402474 PMC12253066

[9] Nakajima T, Harashima S, Hirata M, Kajitani T. Prognostic and therapeutic values of peritoneal cytology in gastric cancer. Acta Cytol 1978;22: 225–229. 281825

[10] Ishii T, Fujiwara Y, Ohnaka S, Hayashi T, Taniguchi H, Takiguchi S, et al Rapid genetic diagnosis with the transcription-reverse transcription concerted reaction system for cancer micrometastasis. Ann Surg Oncol 2004;11: 778–785. 15289240 10.1245/ASO.2004.12.043

[11] Ito S, Nakanishi H, Kodera Y, Mochizuki Y, Tatematsu M, Yamamura Y. Prospective validation of quantitative CEA mRNA detection in peritoneal washes in gastric carcinoma patients. Br J Cancer 2005;93: 986–992. 16205696 10.1038/sj.bjc.6602802 PMC2361668

[12] Katsuragi K, Yashiro M, Sawada T, Osaka H, Ohira M, Hirakawa K. Prognostic impact of PCR-based identification of isolated tumour cells in the peritoneal lavage fluid of gastric cancer patients who underwent a curative R0 resection. Br J Cancer 2007;97: 550–556. 17667927 10.1038/sj.bjc.6603909 PMC2360343

[13] Yonemura Y, Fujimura T, Ninomiya I, Kim BS, Bandou E, Sawa T, et al Prediction of peritoneal micrometastasis by peritoneal lavaged cytology and reverse transcriptase-polymerase chain reaction for matrix metalloproteinase-7 mRNA. Clin Cancer Res 2001;7: 1647–1653. 11410502

[14] Xiao Y, Zhang J, He X, Ji J, Wang G. Diagnostic values of carcinoembryonic antigen in predicting peritoneal recurrence after curative resection of gastric cancer: a meta-analysis. Ir J Med Sci 2014;183: 557–564. 10.1007/s11845-013-1051-6 24378872

[15] Pecqueux M, Fritzmann J, Adamu M, Thorlund K, Kahlert C, Reissfelder C, et al Free intraperitoneal tumor cells and outcome in gastric cancer patients: a systematic review and meta-analysis. Oncotarget 2015;6: 35564–35578. 10.18632/oncotarget.5595 26384352 PMC4742125

[16] Hiraki M, Kitajima Y, Sato S, Nakamura J, Hashiguchi K, Noshiro H, et al Aberrant gene methylation in the peritoneal fluid is a risk factor predicting peritoneal recurrence in gastric cancer. World J Gastroenterol 2010;16: 330–338. 20082478 10.3748/wjg.v16.i3.330 PMC2807953

[17] Kodera Y, Nakanishi H, Ito S, Mochizuki Y, Yamamura Y, Fujiwara M, et al Detection of disseminated cancer cells in linitis plastica-type gastric carcinoma. Jpn J Clin Oncol 2004;34: 525–531. 15466826 10.1093/jjco/hyh097

[18] Kodera Y, Nakanishi H, Ito S, Yamamura Y, Fujiwara M, Koike M, et al Prognostic significance of intraperitoneal cancer cells in gastric carcinoma: detection of cytokeratin 20 mRNA in peritoneal washes, in addition to detection of carcinoembryonic antigen. Gastric Cancer 2005;8: 142–148. 16086116 10.1007/s10120-005-0318-7

[19] Kodera Y, Nakanishi H, Ito S, Yamamura Y, Kanemitsu Y, Shimizu Y, et al Quantitative detection of disseminated cancer cells in the greater omentum of gastric carcinoma patients with real-time RT-PCR: a comparison with peritoneal lavage cytology. Gastric Cancer 2002;5: 69–76. 12111581 10.1007/s101200200012

[20] Kodera Y, Nakanishi H, Ito S, Yamamura Y, Kanemitsu Y, Shimizu Y, et al Quantitative detection of disseminated free cancer cells in peritoneal washes with real-time reverse transcriptase-polymerase chain reaction: a sensitive predictor of outcome for patients with gastric carcinoma. Ann Surg 2002;235: 499–506. 11923605 10.1097/00000658-200204000-00007 PMC1422464

[21] Kodera Y, Nakanishi H, Yamamura Y, Shimizu Y, Torii A, Hirai T, et al Prognostic value and clinical implications of disseminated cancer cells in the peritoneal cavity detected by reverse transcriptase-polymerase chain reaction and cytology. Int J Cancer 1998;79: 429–433. 9699538 10.1002/(sici)1097-0215(19980821)79:4<429::aid-ijc20>3.0.co;2-z

[22] Mori K, Aoyagi K, Ueda T, Danjoh I, Tsubosa Y, Yanagihara K, et al Highly specific marker genes for detecting minimal gastric cancer cells in cytology negative peritoneal washings. Biochem Biophys Res Commun 2004;313: 931–937. 14706632 10.1016/j.bbrc.2003.12.025

[23] Yonemura Y, Endou Y, Fujimura T, Fushida S, Bandou E, Kinoshita K, et al Diagnostic value of preoperative RT-PCR-based screening method to detect carcinoembryonic antigen-expressing free cancer cells in the peritoneal cavity from patients with gastric cancer. ANZ J Surg 2001;71: 521–528. 11527261 10.1046/j.1440-1622.2001.02187.x

[24] Hiraki M, Kitajima Y, Koga Y, Tanaka T, Nakamura J, Hashiguchi K, et al Aberrant gene methylation is a biomarker for the detection of cancer cells in peritoneal wash samples from advanced gastric cancer patients. Ann Surg Oncol 2011;18: 3013–3019. 10.1245/s10434-011-1636-0 21409489

[25] Kodera Y, Nakanishi H, Ito S, Mochizuki Y, Ohashi N, Yamamura Y, et al Prognostic significance of intraperitoneal cancer cells in gastric carcinoma: analysis of real time reverse transcriptase-polymerase chain reaction after 5 years of followup. J Am Coll Surg 2006;202: 231–236. 16427547 10.1016/j.jamcollsurg.2005.09.008

[26] Mori K, Suzuki T, Uozaki H, Nakanishi H, Ueda T, Matsuno Y, et al Detection of minimal gastric cancer cells in peritoneal washings by focused microarray analysis with multiple markers: clinical implications. Ann Surg Oncol 2007;14: 1694–1702. 17294072 10.1245/s10434-006-9321-4

[27] ChangQing F, Yi L, DeGuang W, QingBin S, XiangMin H, Na T, et al Immune clearance gastric carcinoma cells in ascites by activating caspase-9-induced apoptosis. APMISAPMIS 2011;119: 173–179. 10.1111/j.1600-0463.2010.02707.x 21284734

[28] Han J, Lv P, Yu JL, Wu YC, Zhu X, Hong LL, et al Circulating methylated MINT2 promoter DNA is a potential poor prognostic factor in gastric cancer. Dig Dis Sci 2014;59: 1160–1168. 10.1007/s10620-013-3007-0 24385013

[29] Jeon CH, Shin IH, Park JB, Chae HD. Prognostic significance of MAGE in peritoneal washes in gastric carcinoma patients without peritoneal metastasis: results of a 5-year follow-up study. J Clin Gastroenterol 2010;44: 682–686. 10.1097/MCG.0b013e3181d6bb0b 20421806

[30] Yu JL, Lv P, Han J, Zhu X, Hong LL, Zhu WY, et al Methylated TIMP-3 DNA in body fluids is an independent prognostic factor for gastric cancer. Arch Pathol Lab Med 2014;138: 1466–1473. 10.5858/arpa.2013-0285-OA 25357107

[31] Parmar MK, Torri V, Stewart L. Extracting summary statistics to perform meta-analyses of the published literature for survival endpoints. Stat Med 1998;17: 2815–2834. 9921604 10.1002/(sici)1097-0258(19981230)17:24<2815::aid-sim110>3.0.co;2-8

[32] Wells GA, Shea B, O'Connell D, Peterson J, Welch V, Losos M, et al. The Newcastle-Ottawa Scale (NOS) for assessing the quality of nonrandomised studies in meta-analyses. Available:http://www.ohri.ca/programs/clinical_epidemiology/oxford.asp. 2012: Accessed 26 December 2012.

[33] Lau J, Ioannidis JP, Schmid CH. Quantitative synthesis in systematic reviews. Ann Intern Med 1997;127: 820–826. 9382404 10.7326/0003-4819-127-9-199711010-00008

[34] MANTEL N, HAENSZEL W. Statistical aspects of the analysis of data from retrospective studies of disease. J Natl Cancer Inst 1959;22: 719–748. 13655060

[35] DerSimonian R, Laird N. Meta-analysis in clinical trials. Control Clin Trials 1986;7: 177–188. 3802833 10.1016/0197-2456(86)90046-2

[36] Egger M, Davey SG, Schneider M, Minder C. Bias in meta-analysis detected by a simple, graphical test. BMJ 1997;315: 629–634. 9310563 10.1136/bmj.315.7109.629 PMC2127453

[37] Fujiwara Y, Okada K, Hanada H, Tamura S, Kimura Y, Fujita J, et al The clinical importance of a transcription reverse-transcription concerted (TRC) diagnosis using peritoneal lavage fluids in gastric cancer with clinical serosal invasion: a prospective, multicenter study. Surgery 2014;155: 417–423. 10.1016/j.surg.2013.10.004 24439740

[38] Yoneda A, Taniguchi K, Torashima Y, Susumu S, Kanetaka K, Kuroki T, et al The detection of gastric cancer cells in intraoperative peritoneal lavage using the reverse transcription—loop-mediated isothermal amplification method. J Surg Res 2014;187: e1–6. 10.1016/j.jss.2013.01.001 24360119

[39] Satoh Y, Mori K, Kitano K, Kitayama J, Yokota H, Sasaki H, et al Analysis for the combination expression of CK20, FABP1 and MUC2 is sensitive for the prediction of peritoneal recurrence in gastric cancer. Jpn J Clin Oncol 2012;42: 148–152. 10.1093/jjco/hyr179 22172348

[40] Okada K, Fujiwara Y, Nakamura Y, Takiguchi S, Nakajima K, Miyata H, et al Oncofetal protein, IMP-3, a potential marker for prediction of postoperative peritoneal dissemination in gastric adenocarcinoma. J Surg Oncol 2012;105: 780–785. 10.1002/jso.22108 22012575

[41] Tamura N, Iinuma H, Takada T. Prospective study of the quantitative carcinoembryonic antigen and cytokeratin 20 mRNA detection in peritoneal washes to predict peritoneal recurrence in gastric carcinoma patients. Oncol Rep 2007;17: 667–672. 17273749

[42] Fukumoto Y, Ikeguchi M, Matsumoto S, Inoue M, Osaki T, Fukuda K, et al Detection of cancer cells and gene expression of cytokines in the peritoneal cavity in patients with gastric cancer. Gastric Cancer 2006;9: 271–276. 17235628 10.1007/s10120-006-0390-7

[43] Oyama K, Terashima M, Takagane A, Maesawa C. Prognostic significance of peritoneal minimal residual disease in gastric cancer detected by reverse transcription-polymerase chain reaction. Br J Surg 2004;91: 435–443. 15048743 10.1002/bjs.4455

[44] Nakanishi H, Kodera Y, Yamamura Y, Kuzuya K, Nakanishi T, Ezaki T, et al Molecular diagnostic detection of free cancer cells in the peritoneal cavity of patients with gastrointestinal and gynecologic malignancies. Cancer Chemother Pharmacol 1999;43 Suppl: S32–36. 10357556 10.1007/s002800051095

[45] Takebayashi K, Murata S, Yamamoto H, Ishida M, Yamaguchi T, Kojima M, et al Surgery-induced peritoneal cancer cells in patients who have undergone curative gastrectomy for gastric cancer. Ann Surg Oncol 2014;21: 1991–1997. 10.1245/s10434-014-3525-9 24499832

[46] Horikawa M, Iinuma H, Inoue T, Ogawa E, Fukushima R. Clinical significance of intraperitoneal CD44 mRNA levels of magnetically separated CD45-negative EpCAM-positive cells for peritoneal recurrence and prognosis in stage II and III gastric cancer patients. Oncol Rep 2011;25: 1413–1420. 10.3892/or.2011.1191 21331451

[47] Yabusaki N. Clinical significance of zinc-finger E-box binding homeobox 1 mRNA levels in peritoneal washing for gastric cancer. Molecular and Clinical OncologyMol. Clin. Oncol. 2015;3: 435–441. 10.3892/mco.2014.462 PMC4360855 25798282

[48] Takata A. Prognostic value of CEA and CK20 mRNA in the peritoneal lavage fluid of patients undergoing curative surgery for gastric cancer. World journal of surgeryWorld J Surg 2014;38: 1107–1111. 10.1007/s00268-013-2385-y 24305936

[49] Hara M, Nakanishi H, Jun Q, Kanemitsu Y, Ito S, Mochizuki Y, et al Comparative analysis of intraperitoneal minimal free cancer cells between colorectal and gastric cancer patients using quantitative RT-PCR: possible reason for rare peritoneal recurrence in colorectal cancer. Clin Exp Metastasis 2007;24: 179–189. 17487561 10.1007/s10585-007-9067-9

[50] Jeon CH, Kim IH, Chae HD. Prognostic value of genetic detection using CEA and MAGE in peritoneal washes with gastric carcinoma after curative resection: result of a 3-year follow-up. Medicine (Baltimore) 2014;93: e83. 25192488 10.1097/MD.0000000000000083 PMC4616273

[51] Li Z, Zhang D, Zhang H, Miao Z, Tang Y, Sun G, et al Prediction of peritoneal recurrence by the mRNA level of CEA and MMP-7 in peritoneal lavage of gastric cancer patients. Tumour Biol 2014;35: 3463–3470. 10.1007/s13277-013-1458-8 24282089

[52] Miyagawa K, Sakakura C, Nakashima S, Yoshikawa T, Fukuda K, Kin S, et al Overexpression of RegIV in peritoneal dissemination of gastric cancer and its potential as A novel marker for the detection of peritoneal micrometastasis. Anticancer Res 2008;28: 1169–1179. 18505053

[53] Wong J, Kelly KJ, Mittra A, Gonen M, Allen P, Fong Y, et al Rt-PCR increases detection of submicroscopic peritoneal metastases in gastric cancer and has prognostic significance. J Gastrointest Surg 2012;16: 889–896. 10.1007/s11605-012-1845-2 22362071

[54] Tamura S, Fujiwara Y, Kimura Y, Fujita J, Imamura H, Kinuta M, et al Prognostic information derived from RT-PCR analysis of peritoneal fluid in gastric cancer patients: results from a prospective multicenter clinical trial. J Surg Oncol 2014;109: 75–80. 10.1002/jso.23472 24155213

[55] Wang JY, Lin SR, Lu CY, Chen CC, Wu DC, Chai CY, et al Gastric cancer cell detection in peritoneal lavage: RT-PCR for carcinoembryonic antigen transcripts versus the combined cytology with peritoneal carcinoembryonic antigen levels. Cancer Lett 2005;223: 129–135. 15890245 10.1016/j.canlet.2004.09.031

[56] Fujii S, Kitayama J, Kaisaki S, Sasaki S, Seto Y, Tominaga O, et al Carcinoembryonic antigen mRNA in abdominal cavity as a useful predictor of peritoneal recurrence of gastric cancer with serosal exposure. J Exp Clin Cancer Res 2002;21: 547–553. 12636101

[57] Nakabayashi K. Rapid detection of CEA mRNA in peritoneal washes using One-Step Nucleic acid Amplification (OSNA(registered trademark)) for gastric cancer patients. Clinica Chimica ActaClin. Chim. Acta 2015;439: 137–142. 10.1016/j.cca.2014.10.014 25454718

[58] Tokuda K, Natsugoe S, Nakajo A, Miyazono F, Ishigami S, Hokita S, et al Clinical significance of CEA-mRNA expression in peritoneal lavage fluid from patients with gastric cancer. Int J Mol Med 2003;11: 79–84. 12469223

[59] Lee SR, Kim HO, Shin JH, Yoo CH. Prognostic significance of quantitative carcinoembryonic antigen and cytokeratin 20 mRNA detection in peritoneal washes of gastric cancer patients. Hepatogastroenterology 2013;60: 1237–1244. 23321122 10.5754/hge121058

[60] Rossi DMS, Ranieri D, Mazzetta F, Kazemi NA, Raffa S, Torrisi MR, et al Free peritoneal tumor cells detection in gastric and colorectal cancer patients. J Surg Oncol 2012;106: 17–23. 10.1002/jso.23052 22258756

[61] Sugita Y, Fujiwara Y, Taniguchi H, Mori T, Ishii T, Niwa H, et al Quantitative molecular diagnosis of peritoneal lavage fluid for prediction of peritoneal recurrence in gastric cancer. Int J Oncol 2003;23: 1419–1423. 14532985 10.3892/ijo.23.5.1419

[62] Coccolini F, Cotte E, Glehen O, Lotti M, Poiasina E, Catena F, et al Intraperitoneal chemotherapy in advanced gastric cancer. Meta-analysis of randomized trials. Eur J Surg Oncol 2014;40: 12–26. 10.1016/j.ejso.2013.10.019 24290371

