# Supplementary material for: Prognostic Significance of Molecular Analysis of Peritoneal Fluid for Patients with Gastric Cancer: A Meta-Analysis
Source: PLoS One. 2016 Mar 17;11(3):e0151608. doi: 10.1371/journal.pone.0151608 (PMC4795629; doi:10.1371/journal.pone.0151608)
Supplement: S1 Fig — (DOC) [file pone.0151608.s001.doc]

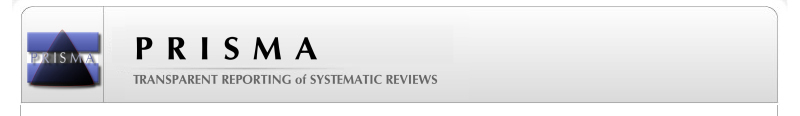
**PRISMA 2009 Flow Diagram**

**Screening**

**Included**

**Eligibility**

**Identification**

Records identified through database searching
(n = 158)

Additional records identified through other sources
(n = 16)

Records after duplicates removed
(n = 58)

Records screened
(n = 116 )

Records excluded
(n = 8)

Full-text articles assessed for eligibility
(n = 108)

Full-text articles excluded, with reasons
(n = 77)

Studies included in qualitative synthesis
(n = 31)

Studies included in quantitative synthesis (meta-analysis)
(n = 31)
