# Supplementary material for: Prognostic Significance of Molecular Analysis of Peritoneal Fluid for Patients with Gastric Cancer: A Meta-Analysis
Source: PLoS One. 2016 Mar 17;11(3):e0151608. doi: 10.1371/journal.pone.0151608 (PMC4795629; doi:10.1371/journal.pone.0151608)
Supplement: S1 File — (DOC) [file pone.0151608.s002.doc]

**File S1. Detailed explanation for extraction of HR**

**Extracting HRs and 95% CIs from Survival Analyses in the Literature for Meta-Analysis**

In the current study, unavailable HRs and 95% CIs were estimated using a previously reported method^[1]^, as detailed below.

**1. Direct estimation of the log hazard ratio and its variance** ^[1]^

For each trial, two methods were used to directly estimate the log hazard ratio, ln(HR_i_), and its 95% CI:

$\ln(HR_{i})=\ln\left(\frac{O_{ri}/E_{ri}}{O_{ci}/E_{ci}}\right)$, $Var[\ln(HR_{i})]=\left[\left(\frac{1}{E_{ri}}\right)+\left(\frac{1}{E_{ci}}\right)\right]$

Or

$\ln(HR_{i})=\left(\frac{O_{ri}-E_{ri}}{V_{ri}}\right)$, $Var[\ln(HR_{i})]=1/V_{ri}$

where O_ri_ = observed number of events in the research group,

O_ci_ = observed number of events in the control group,

E_ri_ = log-rank expected number of events in the treated group,

E_ci_ = log-rank expected number of events in the control group,

Vri = Mantel-Haenszel variance for the log-rank test,

1/Vri = Mantel-Haenszel variance of the log hazard ratio,

Var[ln(HR_i_)] = variance for the log hazard ratio, and

$\Phi$ is the cumulative distribution function of the normal distribution.

$\Phi^{-1}(1-p_{i}/2)$ will equal 1.96 at a two-sided significance of 0.05.

$UPPCI_{i}$ and $LOWCI_{i}$ respectively represent the upper and lower limits of the confidence interval for $\ln(HR_{i})$.

**Estimating HR and 95% CI**

$$HR_{i}=e^{\ln(HR_{i})}$$

$$LOWCI_{i}=\ln(HR_{i})-1.96\times\sqrt{Var[\ln(HR_{i})]}$$

$$UPPCI_{i}=\ln(HR_{i})+1.96\times\sqrt{Var[\ln(HR_{i})]}$$

95% CI: $e^{LOWCI_{i}=\ln(HR_{i})-1.96\times\sqrt{Var[\ln(HR_{i})]}}$ ~ $e^{UPPCI_{i}=\ln(HR_{i})+1.96\times\sqrt{Var[\ln(HR_{i})]}}$

**2. Indirect estimation of the log hazard ratio and its variance indirectly**

$Var[\ln(HR_{i})]=\left[\frac{UPPCI_{i}-LOWCI_{i}}{\Phi^{-1}(1-p_{i}/2)}\right]$, $\ln(HR_{i})=\left(UPPCI_{i}+LOWCI_{i}\right)/2$

UPPCIi and LOWCIi are the respective upper and lower limits of the confidence interval for $\ln(HR_{i})$.

$\Phi^{-1}(1-p_{i}/2)$ will equal 1.96 at a two-sided significance of 0.05.

**Estimating HR and 95% CI**

$$HR_{i}=e^{\ln(HR_{i})}$$

$$LOWCI_{i}=\ln(HR_{i})-\Phi^{-1}(1-p_{i}/2)\times\sqrt{Var[\ln(HR_{i})]}$$

$$UPPCI_{i}=\ln(HR_{i})+\Phi^{-1}(1-p_{i}/2)\times\sqrt{Var[\ln(HR_{i})]}$$

95% CI: $e^{LOWCI_{i}=\ln(HR_{i})-1.96\times\sqrt{Var[\ln(HR_{i})]}}$ ~ $e^{UPPCI_{i}=\ln(HR_{i})+1.96\times\sqrt{Var[\ln(HR_{i})]}}$

**Indirect estimation of the log hazard ratio and 95%CI using the log-rank test**^[1]^

$$\left[\frac{\ln(HR_{i})}{\sqrt{Var[\ln(HR_{i})])}}\right]^{2}=\chi_{\Phi^{-1}(1-p_{i}/2)}^{2}$$

$p_{i}$ = the *p* value of the log-rank test in the ith ($i=1,2,3,\cdots n$) study.

$\chi_{\Phi^{-1}(1-p_{i}/2)}^{2}$ = $\chi^{2}$ of the log-rank test reported in the ith study.

$$\frac{O_{ri}-E_{ri}}{\sqrt{V_{ri}}}=\sqrt{\chi_{\Phi^{-1}(1-p_{i}/2)}^{2}}$$

Two reasonable estimates of $V_{ri}$ are shown as follows:^[2, 3]^

$V_{ri}\approx\frac{O_{i}}{4}$ or $V_{ri}\approx\frac{O_{ri}O_{ci}}{O_{i}}$ $Var[\ln(HR_{i})]=1/V_{ri}$

**Estimating HR and 95% CI**

$$HR_{i}=e^{\ln(HR_{i})}$$

$$LOWCI_{i}=\ln(HR_{i})-\Phi^{-1}(1-p_{i}/2)\times\sqrt{Var[\ln(HR_{i})]}$$

$$UPPCI_{i}=\ln(HR_{i})+\Phi^{-1}(1-p_{i}/2)\times\sqrt{Var[\ln(HR_{i})]}$$

95% CI: $e^{LOWCI_{i}=\ln(HR_{i})-1.96\times\sqrt{Var[\ln(HR_{i})]}}$ ~ $e^{UPPCI_{i}=\ln(HR_{i})+1.96\times\sqrt{Var[\ln(HR_{i})]}}$

$O_{i}$ = the total number of deaths in the two groups.

**Estimation of the log hazard ratio and its 95% CI from survival curves**^[1, 4]^

$R_{ri}(t)=R_{ri}(t-1)-D_{ri}(t-1)-C_{ri}(t)$, $R_{ci}(t)=R_{ci}(t-1)-D_{ci}(t-1)-C_{ci}(t)$;

$R_{ri}(t_{s})=R_{ri}(t-1)-D_{ri}(t-1)$, $R_{ci}(t_{s})=R_{ci}(t-1)-D_{ci}(t-1)$;

$R_{ri}(t)=R_{ri}(t_{s})-C_{ri}(t)$, $R_{ci}(t)=R_{ci}(t_{s})-C_{ci}(t)$;

$D_{ri}(t)=\left[R_{ri}(t)\times\left(\frac{S_{ri}(t_{s})-S_{ri}(t_{e})}{S_{ri}(t_{s})}\right)\right]$, $D_{ci}(t)=\left[R_{ci}(t)\times\left(\frac{S_{ci}(t_{s})-S_{ci}(t_{e})}{S_{ci}(t_{s})}\right)\right]$;

When time to an event and censoring are not formally included in the calculation, then the hazard ratio can be estimated by the relative risk^[4]^.

$\ln\left(HRi(t)\right)=\ln\left(\frac{D_{ri}(t)/R_{ri}(t)}{D_{ci}(t)/R_{ci}(t)}\right)$;

$Var\left[\ln(HR_{i}(t))\right]=\frac{1}{D_{ri}(t)}-\frac{1}{R_{ri}(t)}+\frac{1}{D_{ci}(t)}-\frac{1}{R_{ci}(t)}$;

$\ln(HR_{i})=\frac{\sum_{i=1}^{T} \frac{\ln(HR_{i}(t))}{Var[\ln(HR_{i}(t))]}}{\sum_{i=1}^{T} \frac{1}{Var[\ln(HR_{i}(t))]}}$;$Var[\ln(HR_{i})]=\left[\sum\frac{1}{Var\left[\ln(HR_{i}(t))\right]}\right]^{-1}$;

$R_{ri}(t)$, $R_{ci}(t)$, $R_{ri}(t-1)$ and $R_{ci}(t-1)$ are the effective numbers of patients at risk in the research and control groups during time interval (t-1, t), (t-2, t-1).

$R_{ri}(t_{s})$ and $R_{ci}(t_{s})$ are the effective numbers of patients alive and at risk at the start of the time interval (t-1, t).

$D_{ri}(t)$, $D_{ci}(t)$, $D_{ri}(t-1)$ and $D_{ci}(t-1)$ are the effective numbers of deaths in the research and control groups during time interval (t-1, t), (t-2, t-1).

$C_{ri}(t)$ and $C_{ci}(t)$ are the effective numbers of patients censored in the research and control groups during time interval (t-1, t).

$S_{ri}(t_{s})$ and $S_{ci}(t_{s})$ are the estimates of survival probability for the research and control groups at the start of time interval (t-1, t), read from the Kaplan-Meier curve.

$S_{ri}(t_{e})$ and $S_{ci}(t_{e})$ are the estimates of survival probability for the research and control groups at the end of time interval (t-1, t), read from the Kaplan-Meier curve.

**Estimating HR and 95% CI**

$$HR_{i}=e^{\ln(HR_{i})}$$

$$LOWCI_{i}=\ln(HR_{i})-\Phi^{-1}(1-p_{i}/2)\times\sqrt{Var[\ln(HR_{i})]}$$

$$UPPCI_{i}=\ln(HR_{i})+\Phi^{-1}(1-p_{i}/2)\times\sqrt{Var[\ln(HR_{i})]}$$

95% CI: $e^{LOWCI_{i}=\ln(HR_{i})-1.96\times\sqrt{Var[\ln(HR_{i})]}}$ ~ $e^{UPPCI_{i}=\ln(HR_{i})+1.96\times\sqrt{Var[\ln(HR_{i})]}}$

**References**

[1] Parmar MK, Torri V, Stewart L. Extracting summary statistics to perform meta-analyses of the published literature for survival endpoints. Stat Med. 1998. 17(24): 2815-34.

[2] Kalbßeisch JDaP, R. L. The Statistical Analysis of Failure time Data. Oxford University Press, Oxford,. 1980 .

[3] Tsiatis AA. The asymptotic joint distribution of the e¦cient scores test for the proportional hazards model calculated over time. Biometrika. 1981. 68.

[4] Parmar MKBaM, D. Survival Analysis: Practical Approach. Wiley, Chichester, 1995.. .
