# Supplementary material for: Prognostic Significance of Molecular Analysis of Peritoneal Fluid for Patients with Gastric Cancer: A Meta-Analysis
Source: PLoS One. 2016 Mar 17;11(3):e0151608. doi: 10.1371/journal.pone.0151608 (PMC4795629; doi:10.1371/journal.pone.0151608)
Supplement: S2 Table — (DOC) [file pone.0151608.s004.doc]

**Table S2.** The search results of relevant articles

| Most recent search (2015.11.9) | No. | Searches | Results |
| --- | --- | --- | --- |
| Pubmed | 1# | ("Polymerase Chain Reaction"[All field]) OR ("Reverse Transcriptase Polymerase Chain Reaction"[All field]) | 471764 |
|  | 2# | (minim* resid*) OR ("Flow Cytometry"[Mesh]) OR ("cytology" [Subheading]) OR ("DNA"[Mesh]) OR ("RNA"[Mesh]) OR (shedd* cell*) OR (tumo* cell*) OR (cancer* cell*) OR (neoplas* cell*) | 4956352 |
|  | 3# | ("Stomach Neoplasms"[Mesh] AND English[lang] AND "humans"[Mesh] NOT Case Reports[ptyp] NOT Letter[ptyp] NOT Review[ptyp] NOT Comment[ptyp]) | 33473 |
|  | 4# | (("Ascitic Fluid"[Mesh]) OR (peritone* wash*) OR (peritone* cavi* water) OR (peritoneal* lavage*) OR (efflus*)) | 18939 |
|  | 5# | ((prognos*) OR (risk*) OR (survival*) OR (recurren*) OR (factor*) OR (marker*) OR (biomarker*) OR (relevan*) OR (role*)) | 8114792 |
|  | Total | 1# and 2# and 3# and 4# and 5# | 72 |
| Embase | 1# | prognos* OR risk* OR survival* OR recurren* OR factor* OR marker* OR biomarker* OR relevan* OR role* | 9285635 |
|  | 2# | ascitic* AND fluid* OR (peritone* AND wash*) OR (peritone* AND cavi* AND water) OR (peritoneal* AND lavage*) OR efflus* | 19601 |
|  | 3# | (gastr* OR stomac*) AND (cancer* OR carcinom* OR neoplas* OR tumo*) | 443422 |
|  | 4# | (polymerase AND chain AND reaction) OR (reverse AND transcriptase AND polymerase AND chain AND reaction) AND ((minim* AND resid*) OR (flow AND cytometry) OR cytology OR dna OR rna OR (shedd* AND cell*) OR (tumo* AND cell*) OR (cancer* AND cell*) OR (neoplas* AND cell*)) | 451122 |
|  | 5# | ([article]/lim OR [article in press]/lim OR [conference abstract]/lim OR [conference paper]/lim) AND [english]/lim AND [humans]/lim | 10265087 |
|  | Total | 1# and 2# and 3# and 4# and 5# | 86 |
| Cochrane library | Total | CEA and gastric cancer and survival     search in title, abstract or keyword | 5 |
| JCO and ASCO | Total | Searching gastric cancer and CEA and prognosis (all words) in full text | 11 |
