# Supplementary material for: Prognostic Significance of Molecular Analysis of Peritoneal Fluid for Patients with Gastric Cancer: A Meta-Analysis
Source: PLoS One. 2016 Mar 17;11(3):e0151608. doi: 10.1371/journal.pone.0151608 (PMC4795629; doi:10.1371/journal.pone.0151608)
Supplement: S3 Table — (DOC) [file pone.0151608.s005.doc]

| **Table S3.** Assessment of risk of bias^1^ | | | | | | | |
| --- | --- | --- | --- | --- | --- | --- | --- |
| Study | Were adequate eligibility criteria developed and applied? | Was the measurement of both exposure and outcome adequate? | Was confounding adequately controlled for? | Was the follow-up complete and adequate in duration? | Are reports of the study free of suggestion of selective outcome reporting? | Was the study free of other problems that put it at a high risk of bias? | Risk of bias |
| Fujii S 2002^2^ | Y | Unclear | Unclear | Unclear | Y | Y | N |
| Fujiwara 2014^3^ | Y | Y | Y | Y | Y | Y | Y |
| Fukumoto Y 2006^4^ | Y | Unclear | Unclear | Y | Y | Y | N |
| Hara M 2007^5^ | Y | Y | Y | Y | Y | Y | Y |
| Ishii T 2004^6^ | Y | Y | Y | Y | Y | Y | Y |
| Ito S 2005^7^ | Y | Y | Y | Y | Y | Y | Y |
| Katsuragi K 2007^8^ | Y | Y | Y | Y | Y | Y | Y |
| Kodera Y 2006^9^ | Y | Y | Y | Y | Y | Y | Y |
| Lee SR 2013^10^ | Y | Y | Y | Y | Unclear | Y | N |
| Li Z 2014^11^ | Y | Y | Y | Y | Y | Y | Y |
| Masahiro Horikawa 2011^12^ | Y | Y | Y | Y | Y | Y | N |
| Miyagawa K 2008^13^ | Y | Y | Y | Y | Y | Y | Y |
| Mori K 2007^14^ | Y | Y | Y | Y | Y | Y | Y |
| Nakanishi H 1999^15^ | Unclear | Y | Unclear | Y | Unclear | Y | N |
| Okada K 2012^16^ | Y | Y | Y | Y | Y | Y | Y |
| Oyama K 2004^17^ | Y | Y | Y | Y | Y | Y | Y |
| Rossi Del Monte S 2012^18^ | Y | N | Y | Y | Y | Y | N |
| Satoh Y 2012^19^ | Y | Y | Y | Y | Y | Y | Y |
| Sugita Y 2003^20^ | Y | Unclear | Unclear | Unclear | Y | Y | N |
| Takebayashi K 2014^21^ | Y | Y | Y | Y | Y | Y | N |
| Tamura N 2007^22^ | Y | Y | Y | Y | Y | Y | Y |
| Tamura S 2014^23^ | Y | Y | Y | Y | Y | Y | Y |
| Tokuda K 2003^24^ | Y | Y | Unclear | Y | Y | Y | N |
| Wang JY 2005^25^ | Y | Y | Y | Y | Y | Y | Y |
| Wong J2012^26^ | Y | Unclear | Y | Y | Y | Y | N |
| Jeon CH2014^27^ | Y | Y | Unclear | Y | Y | Y | N |
| Nakabayashi K2015^28^ | Y | Y | Unclear | Y | Y | Unclear | N |
| Takata A2014^29^ | Y | Y | Y | Y | Y | Y | Y |
| Yabusaki N2015^30^ | Y | Y | Y | Y | Y | Y | Y |
| Yoneda A2014^31^ | Y | Unclear | Unclear | Y | Y | Y | Y |
| Yonemura Y2001^32^ | Y | Y | Y | Y | Y | Y | Y |

**References**

1. Higgins JPT, Green S. Cochrane Handbook for Systematic Reviews of Interventions Version 5.1.0 [updated March 2011]. The Cochrane Collaboration .

2. Fujii S, Kitayama J, Kaisaki S, et al. Carcinoembryonic antigen mRNA in abdominal cavity as a useful predictor of peritoneal recurrence of gastric cancer with serosal exposure. J Exp Clin Cancer Res 2002; 21:547-553.

3. Fujiwara Y, Okada K, Hanada H, et al. The clinical importance of a transcription reverse-transcription concerted (TRC) diagnosis using peritoneal lavage fluids in gastric cancer with clinical serosal invasion: a prospective, multicenter study. Surgery 2014; 155:417-423.

4. Fukumoto Y, Ikeguchi M, Matsumoto S, et al. Detection of cancer cells and gene expression of cytokines in the peritoneal cavity in patients with gastric cancer. Gastric Cancer 2006; 9:271-276.

5. Hara M, Nakanishi H, Jun Q, et al. Comparative analysis of intraperitoneal minimal free cancer cells between colorectal and gastric cancer patients using quantitative RT-PCR: possible reason for rare peritoneal recurrence in colorectal cancer. Clin Exp Metastasis 2007; 24:179-189.

6. Ishii T, Fujiwara Y, Ohnaka S, et al. Rapid genetic diagnosis with the transcription-reverse transcription concerted reaction system for cancer micrometastasis. Ann Surg Oncol 2004; 11:778-785.

7. Ito S, Nakanishi H, Kodera Y, et al. Prospective validation of quantitative CEA mRNA detection in peritoneal washes in gastric carcinoma patients. Br J Cancer 2005; 93:986-992.

8. Katsuragi K, Yashiro M, Sawada T, et al. Prognostic impact of PCR-based identification of isolated tumour cells in the peritoneal lavage fluid of gastric cancer patients who underwent a curative R0 resection. Br J Cancer 2007; 97:550-556.

9. Kodera Y, Nakanishi H, Ito S, et al. Prognostic significance of intraperitoneal cancer cells in gastric carcinoma: analysis of real time reverse transcriptase-polymerase chain reaction after 5 years of followup. J Am Coll Surg 2006; 202:231-236.

10. Lee SR, Kim HO, Shin JH, et al. Prognostic significance of quantitative carcinoembryonic antigen and cytokeratin 20 mRNA detection in peritoneal washes of gastric cancer patients. Hepatogastroenterology 2013; 60:1237-1244.

11. Li Z, Zhang D, Zhang H, et al. Prediction of peritoneal recurrence by the mRNA level of CEA and MMP-7 in peritoneal lavage of gastric cancer patients. Tumour Biol 2014; 35:3463-3470.

12. Horikawa M, Iinuma H, Inoue T, et al. Clinical significance of intraperitoneal CD44 mRNA levels of magnetically separated CD45-negative EpCAM-positive cells for peritoneal recurrence and prognosis in stage II and III gastric cancer patients. Oncol Rep 2011; 25:1413-1420.

13. Miyagawa K, Sakakura C, Nakashima S, et al. Overexpression of RegIV in peritoneal dissemination of gastric cancer and its potential as A novel marker for the detection of peritoneal micrometastasis. Anticancer Res 2008; 28:1169-1179.

14. Mori K, Suzuki T, Uozaki H, et al. Detection of minimal gastric cancer cells in peritoneal washings by focused microarray analysis with multiple markers: clinical implications. Ann Surg Oncol 2007; 14:1694-1702.

15. Nakanishi H, Kodera Y, Yamamura Y, et al. Molecular diagnostic detection of free cancer cells in the peritoneal cavity of patients with gastrointestinal and gynecologic malignancies. Cancer Chemother Pharmacol 1999; 43 Suppl:S32-36.

16. Okada K, Fujiwara Y, Nakamura Y, et al. Oncofetal protein, IMP-3, a potential marker for prediction of postoperative peritoneal dissemination in gastric adenocarcinoma. J Surg Oncol 2012; 105:780-785.

17. Oyama K, Terashima M, Takagane A, et al. Prognostic significance of peritoneal minimal residual disease in gastric cancer detected by reverse transcription-polymerase chain reaction. Br J Surg 2004; 91:435-443.

18. Rossi DMS, Ranieri D, Mazzetta F, et al. Free peritoneal tumor cells detection in gastric and colorectal cancer patients. J Surg Oncol 2012; 106:17-23.

19. Satoh Y, Mori K, Kitano K, et al. Analysis for the combination expression of CK20, FABP1 and MUC2 is sensitive for the prediction of peritoneal recurrence in gastric cancer. Jpn J Clin Oncol 2012; 42:148-152.

20. Sugita Y, Fujiwara Y, Taniguchi H, et al. Quantitative molecular diagnosis of peritoneal lavage fluid for prediction of peritoneal recurrence in gastric cancer. Int J Oncol 2003; 23:1419-1423.

21. Takebayashi K, Murata S, Yamamoto H, et al. Surgery-induced peritoneal cancer cells in patients who have undergone curative gastrectomy for gastric cancer. Ann Surg Oncol 2014; 21:1991-1997.

22. Tamura N, Iinuma H, Takada T. Prospective study of the quantitative carcinoembryonic antigen and cytokeratin 20 mRNA detection in peritoneal washes to predict peritoneal recurrence in gastric carcinoma patients. Oncol Rep 2007; 17:667-672.

23. Tamura S, Fujiwara Y, Kimura Y, et al. Prognostic information derived from RT-PCR analysis of peritoneal fluid in gastric cancer patients: results from a prospective multicenter clinical trial. J Surg Oncol 2014; 109:75-80.

24. Tokuda K, Natsugoe S, Nakajo A, et al. Clinical significance of CEA-mRNA expression in peritoneal lavage fluid from patients with gastric cancer. Int J Mol Med 2003; 11:79-84.

25. Wang JY, Lin SR, Lu CY, et al. Gastric cancer cell detection in peritoneal lavage: RT-PCR for carcinoembryonic antigen transcripts versus the combined cytology with peritoneal carcinoembryonic antigen levels. Cancer Lett 2005; 223:129-135.

26. Wong J, Kelly KJ, Mittra A, et al. Rt-PCR increases detection of submicroscopic peritoneal metastases in gastric cancer and has prognostic significance. J Gastrointest Surg 2012; 16:889-896.

27. Jeon CH, Kim IH, Chae HD. Prognostic value of genetic detection using CEA and MAGE in peritoneal washes with gastric carcinoma after curative resection: result of a 3-year follow-up. Medicine (Baltimore) 2014; 93:e83.

28. Nakabayashi K. Rapid detection of CEA mRNA in peritoneal washes using One-Step Nucleic acid Amplification (OSNA(registered trademark)) for gastric cancer patients. Clinica Chimica ActaClin. Chim. Acta 2015; 439:137142.

29. Takata A. Prognostic value of CEA and CK20 mRNA in the peritoneal lavage fluid of patients undergoing curative surgery for gastric cancer. World journal of surgeryWorld J Surg 2014; 38:11071111.

30. Yabusaki N. Clinical significance of zinc-finger E-box binding homeobox 1 mRNA levels in peritoneal washing for gastric cancer. Molecular and Clinical OncologyMol. Clin. Oncol. 2015; 3:435441.

31. Yoneda A, Taniguchi K, Torashima Y, et al. The detection of gastric cancer cells in intraoperative peritoneal lavage using the reverse transcription--loop-mediated isothermal amplification method. J Surg Res 2014; 187:e1-6.

32. Yonemura Y, Fujimura T, Ninomiya I, et al. Prediction of peritoneal micrometastasis by peritoneal lavaged cytology and reverse transcriptase-polymerase chain reaction for matrix metalloproteinase-7 mRNA. Clin Cancer Res 2001; 7:1647-1653.
